# *Botrytis cinerea* uses effective detoxification mechanisms to overcome the plant RBOHD-dependent extracellular oxidative burst

**DOI:** 10.3389/fpls.2026.1969467

**Published:** 2026-10-16

**Authors:** Alejandra Vielba-Fernandez, Lea Sommer, Désirée Ross, Julian Ingelfinger, Thorsten Wachtmeister, Jochen Hecht, Nassim Safari, Stefanie J Müller-Schüssele, Matthias Hahn

**Affiliations:** 1 Plant Pathology, Department of Biology, University of Kaiserslautern-Landau, Kaiserslautern, Germany; 2 Institute of Plant Sciences, Plant RNA Biotechnology, University of Regensburg, Regensburg, Germany; 3 Molecular Botany, Department of Biology, University of Kaiserslautern-Landau, Kaiserslautern, Germany; 4 Genomics & Transcriptomics Labor (BMFZ), Heinrich-Heine-University, Düsseldorf, Germany

**Keywords:** B. cinerea, plant immunity, plant-pathogen interaction, RbohD, reactive oxygen species, ROS detoxification

## Abstract

The necrotrophic fungus *Botrytis cinerea* rapidly kills host tissue with its secreted proteins and metabolites. Necrotrophs can trigger the hypersensitive response, which is accompanied by reactive oxygen species (ROS) production, but it remains unclear whether this ROS burst promotes or restricts *B. cinerea* infection. We have combined Arabidopsis RESPIRATORY BURST OXIDASE HOMOLOG D (RBOHD) mutant analysis, luminol-based *in vivo* assays, and the use of CRISPR/Cas9-generated *B. cinerea* ROS detoxification mutants to analyze the toxic effect of the ROS burst. In addition, RNA-sequencing, transcript analyses and redox imaging were used to study RBOHD-dependent signaling and redox homeostasis. *RBOHD* mutants showed slightly increased susceptibility to *B. cinerea* infection, and the apoplastic ROS burst was found to be dependent on RBOHD. Fungal mutants confirmed the importance of SOD1 and AP1 for ROS detoxification and virulence. Unexpectedly, the transcriptomes of *B. cinerea*-infected Arabidopsis Col-0 and an activation-impaired *RBOHD* mutant were highly similar 48 hours after inoculation, providing no evidence for a connection of extracellular ROS production and intracellular ROS-induced transcriptional signaling. Nevertheless, RBOHD activity was found to be involved in the maintenance of cellular redox homeostasis under phytotoxic stress. Our results indicate a protective role of the RBOHD-dependent oxidative burst against *B. cinerea*, but this can be overcome by the fungus via different ROS detoxification mechanisms.

## Introduction

Plants possess a robust, two-layered immune system that safeguards them against a variety of pathogens. The first layer, called Pattern-Triggered Immunity (PTI), is activated at the cell surface by Pattern Recognition Receptors (PRRs) which recognize Pathogen-Associated Molecular Patterns (PAMPs), such as bacterial flagellin or fungal chitin fragments. PTI leads to a rapid, moderate response, typically sufficient to confer resistance to most pathogens (Trdá et al., 2015). The second layer, Effector-Triggered Immunity (ETI), is activated usually inside the cell by Nucleotide-binding Leucine-rich repeat Receptors (NLRs) which detect effector proteins of specialized pathogens that have overcome PTI, and builds up a much stronger defense (Ngou et al., 2022). ETI often culminates in the Hypersensitive Response (HR), resulting in cell death of the infected tissue, which effectively confines most pathogens and prevents their spread into the tissue (Balint-Kurti, 2019). Recent studies have demonstrated a close connection between PTI and ETI, where PTI components are necessary for complete ETI-mediated resistance, and ETI enhances PTI signaling (Ngou et al., 2021; Yuan et al., 2021).

Among the earliest immune responses is the oxidative burst, which is the rapid release of reactive oxygen species (ROS), such as superoxide (O_2_^•-^) and hydrogen peroxide (H_2_O_2_). A major source of ROS is plasma membrane-bound NADPH oxidases, called RBOH (Respiratory Burst Oxidase Homolog). In *Arabidopsis thaliana* (Arabidopsis), the major pathogen-induced isoform, RBOHD, is activated during PTI as follows: Upon PAMP recognition, many PRRs recruit the coreceptor BRI1-Associated Kinase 1 (BAK1). This interaction activates receptor-like cytoplasmic kinases such as BIK1 (BOTRYTIS-INDUCED KINASE 1), which phosphorylates serine residues in the N-terminal domain of RBOHD to initiate ROS production (Kadota et al., 2014). RBOHD reduces molecular oxygen with NADPH to the superoxide anion radical (O_2_^•-^) in the apoplast, which is rapidly converted to H_2_O_2_, either spontaneously or by extracellular superoxide dismutases (Gill and Tuteja, 2010; Martínez Rivas et al., 2024). Together with O_2_^•-^, H_2_O_2_ functions as a direct antimicrobial agent, and acts as an oxidant in peroxidase-driven cell wall crosslinking or cell wall loosening through hydroxyl radical–mediated cleavage of polysaccharides (Tenhaken, 2014; Martínez Rivas et al., 2024). In addition, it serves as a signaling molecule after uptake into the cytoplasm via Plasma membrane Intrinsic Proteins (PIP) aquaporins, leading to defense gene activation and systemic acquired resistance (Tian et al., 2016; Zhang et al., 2022). Whereas the PTI-induced oxidative burst is detectable as a rapid and transient peak of extracellular H_2_O_2_, accompanied by intracellular redox responses over the course of hours (Arnaud et al., 2023), the ETI-associated ROS burst is a delayed but stronger and longer-lasting response (Weralupitiya et al., 2024). A defensive role of the ROS burst against pathogens has been documented in some reports. Virus-induced silencing of the NADPH oxidase homologs NbrbohA and NbrbohB in *Nicotiana benthamiana* reduced H_2_O_2_ accumulation, delayed hypersensitive cell death, and significantly increased susceptibility to the hemibiotrophic oomycete *Phytophthora infestans* (Yoshioka et al., 2003). In another study, suppression of OsRac1-mediated activation of ROS production through OsRhoB resulted in enhanced susceptibility to the rice blast fungus *Magnaporthe oryzae* (Wong et al., 2007). However, while Arabidopsis *rbohD* and *rbohF* mutants exhibit defects in pathogen-induced ROS production, they showed altered resistance responses to biotrophic pathogens but no clearly increased susceptibility (Torres et al., 2002).

Fungi are also exposed to self-generated ROS, which are produced during mitochondrial electron transport and metabolic reactions. In addition, they possess intracellular superoxide-generating NADPH oxidases, which are essential for normal development (Heller and Tudzynski, 2011; Viefhues et al., 2014). To protect themselves against oxidative damage and to maintain cellular redox equilibrium, fungi have evolved multiple mechanisms (Park and Son, 2024). A major ROS-defense regulator in fungi is the transcription factor AP-1. In the yeast *Saccharomyces cerevisiae*, Yap1 is activated by H_2_O_2_-dependent oxidation, followed by migration into the nucleus and activation of oxidative stress-responsive genes (Toone and Jones, 1999; Wood et al., 2004). Among plant pathogenic fungi, Yap1/AP-1 mutants of *Ustilago maydis*, *M*. *oryzae* and *Alternaria alternata* showed reduced virulence, whereas in *Fusarium graminearum* and *Botrytis cinerea* such mutants were reported to have no effect on infection (Park and Son, 2024; Temme and Tudzynski, 2009). The Cu–Zn superoxide dismutase SOD1 has been found to be important for virulence in *F. graminearum*, *Verticillium dahliae* and *B. cinerea* (Yao et al., 2016; Tian et al., 2021; López-Cruz et al., 2017), but not in the biotrophic pathogen *Claviceps purpurea* (Moore et al., 2002). Fungi contain several groups and isoforms of cytoplasmic and secreted catalases, which detoxify H_2_O_2_ by disproportionation to H_2_O and O_2_, and peroxidases which use external reductants for converting H_2_O_2_ to H_2_O, but a functional analysis is difficult due to their redundancy. Most of these enzymes proved individually dispensable; however, the catalase SsCat2 from *Sclerotinia sclerotiorum* was found to be required for full virulence (Park and Son, 2024; Huang et al., 2021). Apart from detoxification, several fungi actively suppress the plant oxidative burst during infection. For example, the effector NIS1, found in *Colletotrichum* spp. and *M. oryzae*, inhibits the PRR-associated kinases BAK1 and BIK1, thereby preventing NADPH oxidase activation (Irieda et al., 2019). More broadly, *M. oryzae*–rice interactions involve multiple effectors and pathways that modulate ROS dynamics during infection (Kou et al., 2019).

The gray mold fungus *B. cinerea* causes enormous pre- and postharvest damage to many cultivated plants worldwide (Elad et al., 2016). Infection by this necrotrophic, host-killing pathogen is accompanied by extensive secretion of cell wall-degrading enzymes, phytotoxic proteins and metabolites (Bi et al., 2023). In their seminal publication, Govrin and Levine (2000) demonstrated that the HR, while effective against biotrophs, is exploited by necrotrophs to promote infection. They also reported that *B. cinerea* stimulates the plant ROS burst to trigger HR, based on observations that treatments of leaf tissue with exogenous ROS increased *B. cinerea*-induced lesion formation (Govrin and Levine, 2000). In contrast, Torres et al. (2005) provided evidence that, while RBOHD can be activated by exogenous ROS, the ROS burst suppresses cell death in the vicinity of its activation. In another study, Arabidopsis Col-0 and the *rbohD* mutant showed similar susceptibility to infection by *B. cinerea* (Galletti et al., 2008). A clear defense-related role of the ROS burst was documented for abscisic acid-deficient tomato *sitiens* mutants which are highly resistant to *B. cinerea* and show a rapid accumulation of H_2_O_2_ at the infection sites (Asselbergh et al., 2007).

Several ROS-protective systems have been characterized in *B. cinerea*, but their role in protecting the fungus against the host oxidative burst has not been directly investigated. In this study, we investigated the effects of the plant oxidative burst on infection by *B. cinerea*, testing two hypotheses: (i) the extracellular ROS burst protects the plant against *B. cinerea* infection, and (ii) *B. cinerea* induces ROS production to trigger HR-associated cell death for its benefit. In both scenarios, *B. cinerea* must use effective mechanisms to mitigate the potential damage caused by the oxidative burst. To address these hypotheses, we compared the response of Arabidopsis Col-0 and *RBOHD* mutants to *B. cinerea*, and monitored the oxidative burst elicited by *B. cinerea* infection using luminol-based ROS measurements. Our data demonstrate that RBOHD is the major, if not the only, source of the extracellular oxidative burst induced by *B. cinerea* and clearly has a protective role. Furthermore, by generating and testing marker-free single and multiple ROS-detoxification mutants of *B. cinerea*, we found that their impaired ROS tolerance correlated with reduced virulence. Our findings provide further insights into the mechanisms and roles of ROS production and detoxification in a major necrotrophic plant-fungus interaction.

## Materials and methods

### Plant material and growth conditions

Arabidopsis ecotype Col-0 and the mutants *rbohD* (Torres et al., 2002), *RBOHD S39A/S339A/S343A* (*RBOHD^MUT^*) (Kadota et al., 2014), and *bik1* (Veronese et al., 2006), as well as the reporter line Grx1-roGFP2 (Gutscher et al., 2008), were stratified at 4 °C for 32 h and grown under short-day conditions (8 h light/16 h dark, 22 °C) for 5 weeks. *Solanum lycopersicum* (tomato), *Nicotiana benthamiana*, and *Phaseolus vulgaris* (common bean) were cultivated on soil under long-day conditions (14 h light/10 h dark, 23 °C), and used after 6, 5, and 2 weeks, respectively, for infection tests.

### *Botrytis cinerea* strains, their cultivation and mutagenesis

*B. cinerea* strain B05.10 (WT) and the mutants 18x (a multiple knockout mutant lacking the phytotoxic metabolites botrydial and botcinic acid, and 16 phytotoxic proteins; Leisen et al., 2022; Safari et al., 2026), *bmp1* (Doehlemann et al., 2006) and ROS-detoxification mutants (*sod1, ccp1, cat2, ap1, ap1sod1, ap1sod1cat2*) were cultivated on malt-extract (ME) agar at 22 °C under ambient light, as described by Müller et al. (2018). Marker-free gene deletions were generated by CRISPR/Cas9-mediated transformation as described by Leisen et al. (2020, 2022). Briefly, paired sgRNAs flanking each coding sequence (**Supplementary Table S1**) were used for protoplast transformation with the telomere vector pTEL-Fen; transformants were initially selected on fenhexamid, purified, and validated by PCR (**Supplementary Figure S5**) and sequencing for clean, marker-free deletions. Higher-order mutants were obtained by sequential transformation. For each transformation of double and triple mutants, at least three positive transformants were used in preliminary experiments to ensure that they exhibited the same phenotype before one of them was selected for quantitative analysis.

### Infection and growth assays

For infection assays, *B. cinerea* conidia (1×10^5^ spores/mL in Gamborg’s B5 medium + 25 mM glucose) were applied as 20 µL droplets to detached tomato leaves or attached *P. vulgaris* leaves, and to attached Arabidopsis leaves after 0, 4, 6 and 8 h pre-germination in semi-solid (0.3% agar) Gamborg’s B5 medium with 50 mM glucose. For *N. benthamiana*, inoculum (1×10^5^ spores/mL in water) was applied on 6-mm water-agar plugs; 10 µL Gamborg’s B5 + 25 mM glucose was added beneath each plug. Plants were kept in humid boxes at 22 °C under dim light for 48–60 h, and lesion areas were quantified. Lesion area was determined by subtracting the area of the inoculation droplet from the total infected area, measuring only lesions that extended into leaf tissue beyond the droplet. Within each replicate, lesion areas were normalized to those of the control.

For secretome preparation, inoculation droplets above the lesions were collected from inoculated tomato leaves after 48 h as described by Müller et al. (2018). The clarified secretome was ultrafiltered (10 kDa cut-off), and protein concentration adjusted to 2 µg/mL in imaging buffer (10 mM MES, 5 mM KCl, 10 mM CaCl_2_, 10 mM MgCl_2_, pH 5.8).

For ROS manipulation assays, Arabidopsis leaves were infiltrated with H_2_O_2_ (100–300 mM), glucose oxidase (100 U/mL) + glucose (2 mM; Bankar et al., 2009), diphenyleneiodonium chloride (3 µM; DPI), or 3-amino-1,2,4-triazole (2 mM; 3-AT) 1 h before inoculation as previously described.

*In vitro* growth and oxidative-stress tolerance of ROS-detoxification mutants were assessed on ME agar supplemented with menadione (10 µM) or H_2_O_2_ (2.5, 5, 10 mM) at 22 °C under dim light. Sclerotia formation and sporulation were analyzed after 14 d (dark) or 10 d (light). Germination assays used 1×10^5^ spores/mL in 1× ME + 5 mM H_2_O_2_, or Gamborg’s B5 + 50 mM glucose with either 10 mM H_2_O_2_ or 50 µM menadione; germ-tube lengths were measured after 7.5 h.

### ROS, redox, and cell-death assays

Extracellular ROS production was quantified by luminol-based chemiluminescence. Four leaf discs (7 mm) of at least three different plants of Arabidopsis Col-0, *RBOHD^MUT^*, and *bik1* mutants were cut and floated adaxial side up in 96-well plates containing 300 µL imaging buffer for 1 h in darkness before inoculation. *B. cinerea* WT or mutants (18x, *bmp1*, and ROS-detoxification mutants) were pregerminated for 18 h in ME medium and then applied as a single 3 µL droplet (1×10^5^ spores/mL) on the center of the leaf disc. As a negative control, 3 µL of 1× ME medium was applied onto the leaf disc surface. After 6 h, discs were inverted for ROS detection in 75 µL buffer containing 8 µg/mL horseradish peroxidase (HRP, type II) and 20 µM L-012 (FUJIFILM Wako). Luminescence was recorded for 15–18 h at 22 °C with a CLARIOstar Plus plate reader (BMG Labtech). The experiment was repeated at least three times independently. For the secretome-triggered ROS measurements, the secretome of *B. cinerea* WT produced after 48 h on tomato leaves (Müller et al., 2018; Leisen et al., 2022) was added at 1.5 µg/mL (protein concentration) to the imaging buffer after inversion of the discs. Discs before and after ROS measurement were stained with 10 µg/mL calcofluor white for 5 min and imaged on a Zeiss LSM880 confocal microscope (405 nm and 638 nm excitation for calcofluor and chlorophyll autofluorescence, respectively). Cell death was visualized 24 h after infiltration of 1.5 µg/mL secretome by Trypan Blue staining and quantified in ImageJ relative to total leaf area.

For the cytosolic redox changes assay, four leaf discs (7 mm) of at least three different plants of Arabidopsis Grx1-roGFP2 lines (Col-0 and *rbohD* backgrounds) were cut and floated adaxial side up in 96-well plates containing 300 µL imaging buffer overnight in darkness. After incubation, the imaging buffer was exchanged for new buffer and the discs were incubated for an additional hour in the darkness. After this time period, the basal fluorescence was monitored using excitation at 400 ± 5 nm/482 ± 8 nm and emission at 530 ± 20 nm. After five baseline cycles, buffer containing 2 µg/mL *B. cinerea* WT secretome was added; 10 mM dithiothreitol (DTT) or 10 mM H_2_O_2_ provided reduction/oxidation controls. Oxidation degree (OxD) was calculated following Gutscher et al. (2008).

### RNA-seq and quantitative RT-PCR

Three consecutive infection assays were conducted to obtain three biological replicates. In each replicate, 5 plants per Arabidopsis genotype were infected on at least 5 leaves. At 48 h post inoculation (hpi), the leaves of each line were harvested, pooled and immediately frozen in liquid nitrogen. Total RNA from mock-treated and *B. cinerea*-infected Col-0 and *RBOHD^MUT^* leaves was extracted with the RNeasy Plant Mini Kit (QIAGEN), treated with DNase I, and assessed on a Bioanalyzer. Libraries were prepared with the VAHTS Universal V10 RNA-seq Library Prep Kit for Illumina (Vazyme) and sequenced on a NextSeq 2000 (Illumina; paired-end, 150 bp). This yielded 113.5–150.7 million reads per sample (mean 130.7 million).

Read import, quality control, trimming, mapping and differential expression analysis were performed in CLC Genomics Workbench v24.0.1 (QIAGEN). Reads were imported with Illumina High-Throughput Sequencing Import 2.0, checked with QC for Sequencing Reads 0.2 and trimmed with Trim Reads 3.0. They were then mapped to the *A. thaliana* TAIR10 genome (27,655 annotated genes) with RNA-Seq Analysis 2.8. Mock samples had 95.4–97.9% of read pairs mapped. Infected samples had 42.1–65.3% mapped, consistent with the presence of fungal RNA in infected tissue. Of the counted fragments, 94.5–99.0% mapped to annotated genes. All libraries were reverse-stranded (<0.9% of reads on the wrong strand), had <0.4% adapter read-through, and showed no transcript-length coverage bias. Differential expression was tested with Differential Expression for RNA-Seq 2.8. Genes with an FDR-corrected p ≤ 0.05 and |FC| ≥ 1.5 (|log_2_FC| ≥ 0.585) were considered differentially expressed (DEGs). Principal component analysis was performed with PCA for RNA-Seq 1.4 and heat maps were generated with Create Heat Map for RNA-Seq 1.5. GO annotation was taken from the TAIR GAF release of 2025-02-06 (geneontology.org). Raw reads were deposited in NCBI GEO under accession GSE317547.

For additional quantitative comparisons of the genotypes (**Supplementary Figure S9**), the CLC gene-level counts were reanalyzed with PyDESeq2 v0.5.4 (Muzellec et al., 2023; Love et al., 2014), using genes with at least 10 counts in at least three samples (19,375 genes) and the design replicate + genotype + infection + genotype:infection. Gene set enrichment analysis (GSEA) was performed with GSEApy v1.3.1 (Fang et al., 2023) on all genes pre-ranked by sign(log_2_FC) × –log_1__0_(p) of the *RBOHD^MUT^* vs Col-0 comparison at 48 hpi (Subramanian et al., 2005). Gene sets (15–500 genes) were GO biological process terms from the TAIR annotation (GAF release 2025-02-06), propagated to parent terms. We used 2,000 permutations and considered FDR < 0.05 significant.

For time-course analyses, plant infections were performed as described above, but additional leaf samples were collected at 18, 24, 48, and 72 hpi from three independent biological replicates. cDNA was synthesized using the iScript cDNA Synthesis Kit (Bio-Rad), and the qPCR was performed with Biozym Blue S’Green qPCR Kit on a CFX Connect Real-Time PCR Detection System using primers for the Arabidopsis genes *PR1*, *PDF1.2*, *WRKY33*, *PAD3*, *ERF1*, *BIK1*, and *PAD4* (**Supplementary Table S1**); expression values were normalized to the reference gene ACT2, which has been shown to be stably expressed during *B. cinerea* infection (Ingle et al., 2015), and calculated by the Pfaffl method (Pfaffl, 2004). Primer efficiencies were determined for all primer pairs by standard curve analysis using serial dilutions of DNA from the uninfected control sample (1:10 for ACT2; 1:2 for the expression genes). Efficiency, calculated from the slope of Ct plotted against log-transformed dilution factor (E = (10^(–1/slope) – 1) × 100), ranged from 95.48% to 101.65% across all primer pairs (**Supplementary Table S4**).

### Statistical analysis and software

All images were processed and analyzed using ZEN 2.3 (Zeiss) or ImageJ v1.54p (National Institutes of Health, Bethesda, MD, USA)^1^. Statistical analyses were performed with GraphPad Prism v10.1.1 (GraphPad Software, San Diego, CA, USA). The specific statistical tests applied are indicated in the respective figure legends. Figures were assembled in Inkscape v1.4. The additional RNA-seq analyses were performed in Python (PyDESeq2, GSEApy, scikit-learn, matplotlib).

## Results

### Pretreatment of Arabidopsis leaves with ROS does not increase *B. cinerea* infection

Previously, Govrin and Levine (2000) have reported that pretreatments with ROS or ROS-generating compounds increased *B. cinerea* infection and the hypersensitive response (HR). Specifically, infiltration of leaves prior to infection with a glucose oxidase/glucose mixture, which generates H_2_O_2_ (Wu et al., 1995), increased lesion formation, and similar effects were observed following infiltration with the catalase inhibitor 3-AT. In contrast, infiltration with the NADPH oxidase inhibitor DPI restricted necrosis. To reevaluate their results, we performed similar experiments by infiltrating solutions with ROS-modulating effects into Arabidopsis leaves, including treatments with different concentrations of H_2_O_2_, followed by inoculation with *B. cinerea* WT conidia. Except for the highest concentration of H_2_O_2_ (300 mM), which caused an increased lesion area after infection, none of the treatments resulted in significantly changed lesion formation (**Supplementary Figure S1**). Although the inhibitors 3-AT and DPI are not very specific, and non-physiological concentrations of H_2_O_2_ were used, these results indicate that, in contrast to the data of Govrin and Levine (2000), *B. cinerea* infection is not strongly affected by changes in apoplastic ROS.

### Arabidopsis RBOHD mutants exhibit increased susceptibility to *B. cinerea* infection

In a previous study, no differences were observed between Arabidopsis Col-0 and a *rbohD* knockout mutant in their susceptibility to infection by *B. cinerea* (Galletti et al., 2008). To reevaluate these data and to investigate the role of RBOHD-generated ROS, the same Arabidopsis *rbohD* mutant was used for inoculation with three developmental stages of *B. cinerea*: conidia preincubated for 8 h (fully germinated with long germ tubes), conidia preincubated for 4 h (ca. 50% conidia germinated with short germ tubes), and ungerminated conidia (**Supplementary Figure S2A**). With all inoculum variants, the *rbohD* mutant was slightly but significantly more susceptible to infection. Furthermore, the difference in susceptibility between Col-0 and *rbohD* plants against *B. cinerea* infection increased as pregermination time decreased, and was highest for inoculation with ungerminated conidia (**Supplementary Figures S2B, C**). The *rbohD* mutant used in this study has been described by Torres et al. (2002) and shown to contain an insertion of dSpm transposon in codon 535, leading to truncation and loss of function of RBOHD. This mutant has been reported to show increased levels of salicylic acid and ethylene, and overexpression of the defense marker gene *PR-1* following pathogen infection (Chaouch et al., 2012; Pogány et al., 2009). To assess whether these potentially pleiotropic effects could interfere with functional analysis of RBOHD, and because the *rbohD* mutant showed weaker growth than Col-0, the infection tests were repeated with the mutant *RBOHD S39A/S339A/S343A* (hereafter referred to as *RBOHD^MU^*^T^), in which three serine residues phosphorylated by BIK1 were replaced by alanine, which strongly impairs BIK1-dependent activation of RBOHD (Kadota et al., 2014; Berlanga et al., 2024). Plants were inoculated with fully germinated spores preincubated for 6 h and with ungerminated *B. cinerea* spores (**Figure 1**). The results were similar to those with the *rbohD* mutant: The *RBOHD^MUT^* mutant showed slightly larger lesions than Col-0, and the difference in sensitivity was again more pronounced with ungerminated spores. To compare the sensitivity of pregerminated and ungerminated spores to oxidative stress, spores preincubated for 6 h and ungerminated spores were exposed *in vitro* to 10 mM H_2_O_2_ or 50 µM menadione (which generates superoxide and other ROS species), and germ tube length was measured after 7.5 h. Ungerminated spores did not germinate in the presence of 10 mM H_2_O_2_, and exposure to menadione caused a significant reduction in germ tube length. In pregerminated spores, by contrast, both treatments reduced germ tube elongation only slightly, indicating that ungerminated spores are considerably more sensitive to oxidative stress than pregerminated ones (**Supplementary Figure S3**). These findings demonstrate that the loss or strong impairment of RBOHD activity slightly but significantly increases infection by *B. cinerea*, and that spore germination is the developmental stage which is most sensitive to the oxidative burst.

**Figure 1:**
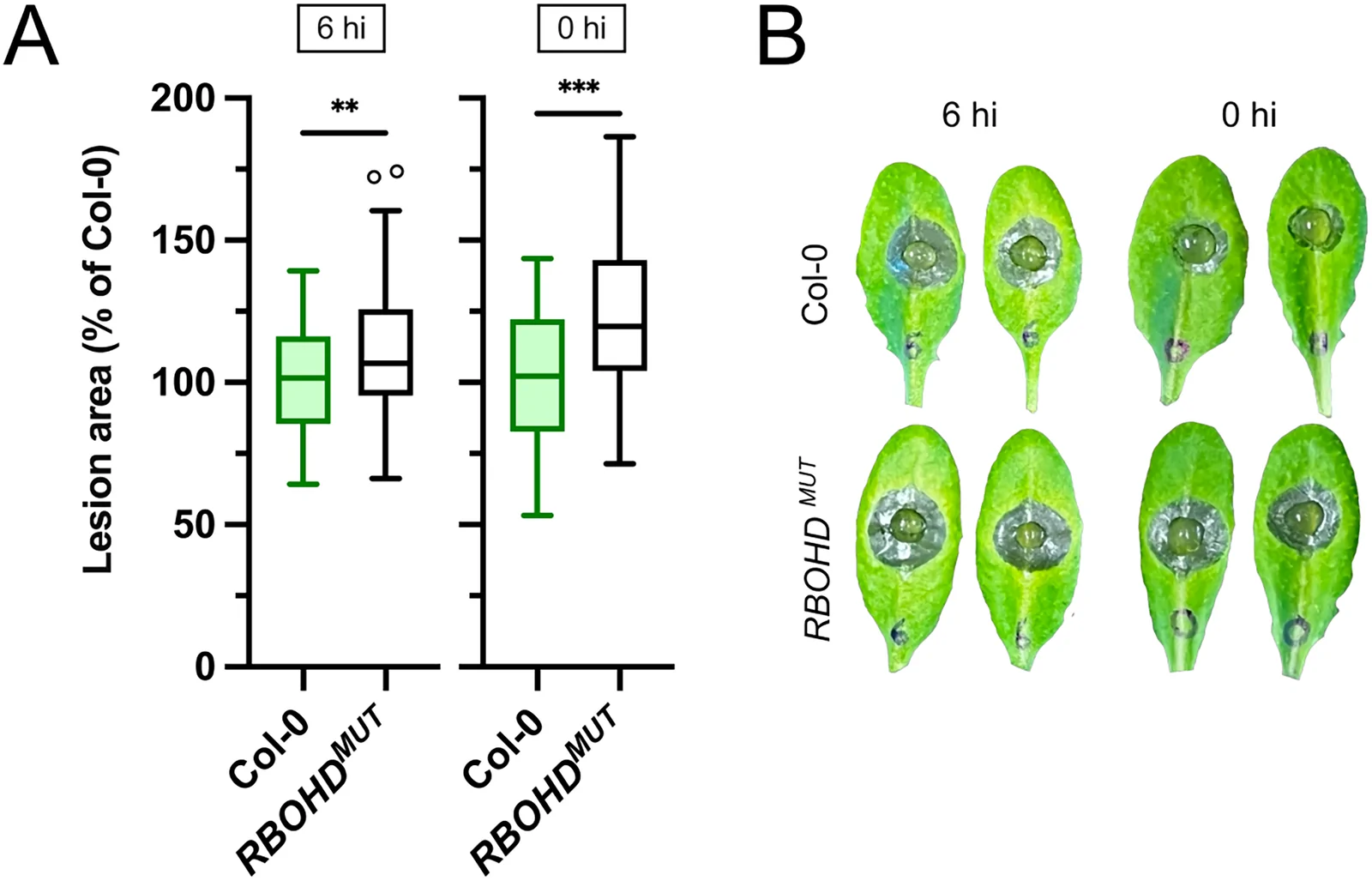
*B. cinerea* infection of Arabidopsis Col-0 and *RBOHD^MUT^.* **(A)** *Box-and-whisker* plots showing lesion sizes in Arabidopsis Col-0 and *RBOHD^MUT^* leaves 48 h after inoculation with *B. cinerea* B05.10 WT spores preincubated for 6 and 0 h. Tests were conducted three times, with six plants per line and at least three infections per plant and strain in each replicate. Raw data and sample sizes are provided in **Supplementary Table S4**. Statistical significance was assessed using an unpaired t-test, **p<0.01, ***p<0.001. hi: hours of preincubation. **(B)** Images of *B. cinerea*-infected Arabidopsis leaves (48 hpi).

### The extracellular ROS burst is triggered during *B. cinerea* penetration and depends on RBOHD

During infection, *B. cinerea* secretes a mixture of proteins containing a large number of lytic enzymes and phytotoxic, cell death-inducing proteins (CDIPs; Leisen et al., 2022). In order to evaluate the role of the phytotoxic secretome for triggering the ROS burst during infection and to follow its kinetics, the release of ROS from Arabidopsis leaf discs in response to treatment with either the secretome or to fungal infection was measured using a luminol-based assay (Jantean et al., 2022; Ugalde et al., 2022) (**Figure 2A**). We also compared the effects caused by the secretomes and by infection of *B. cinerea* WT and an 18x mutant lacking the phytotoxic metabolites botrydial and botcinic acid and 16 CDIPs, which results in strongly reduced phytotoxic activity of its secretome (Safari et al., 2026). While penetration of the 18x mutant into the plant tissue is only weakly reduced, formation of necrotic lesions is strongly delayed compared to WT (Leisen et al., 2022; Müller et al., 2024; Safari et al., 2026). Approximately one hour after addition of the secretomes, a ROS burst lasting for several hours was observed, which was more intense with WT secretome than with the less toxic 18x mutant secretome, but showed similar kinetics (**Figure 2A**). To follow the ROS production in response to *B. cinerea* infection, an assay was established with Arabidopsis leaf discs that were inoculated with pregerminated conidia 6 h before ROS measurements in the luminometer were started. While no measurements were possible during the first 6 h after inoculation, ROS was first detected in the incubation solution 10 h after inoculation with both strains, corresponding to 4 h after the start of measurements. This was followed by a strong increase, which reached its maximum after 6–8 h and then decreased (**Figure 2B**). Unexpectedly, ROS bursts induced by WT and 18x mutant were similar, although the 18x mutant secretome was 3–4 times less toxic than the WT secretome (Safari et al., 2026); both strains gave an area under the curve (AUC) about 12-fold higher than the mock control (**Figure 2B**). To identify the possible trigger of the late ROS response induced by *B. cinerea* infection, a *bmp1* knockout mutant lacking a mitogen-activated protein (MAP) kinase, which is defective in penetration and killing of plant tissue (Doehlemann et al., 2006), was included in the assays. Inoculation of the leaf discs with these strains revealed that the 18x and *bmp1* mutants similarly induced much less killing of plant cells than WT (**Supplementary Figure S4**). In contrast, the ROS release of the leaf discs inoculated with WT and 18x mutant was similar, but much weaker in response to the *bmp1* mutant (**Figure 2B**), with a 3.2-fold lower AUC than WT (**Figure 2B**). These results indicate that the ROS burst in Arabidopsis might be triggered during fungal penetration, and that cell death induction contributes less to ROS accumulation.

**Figure 2:**
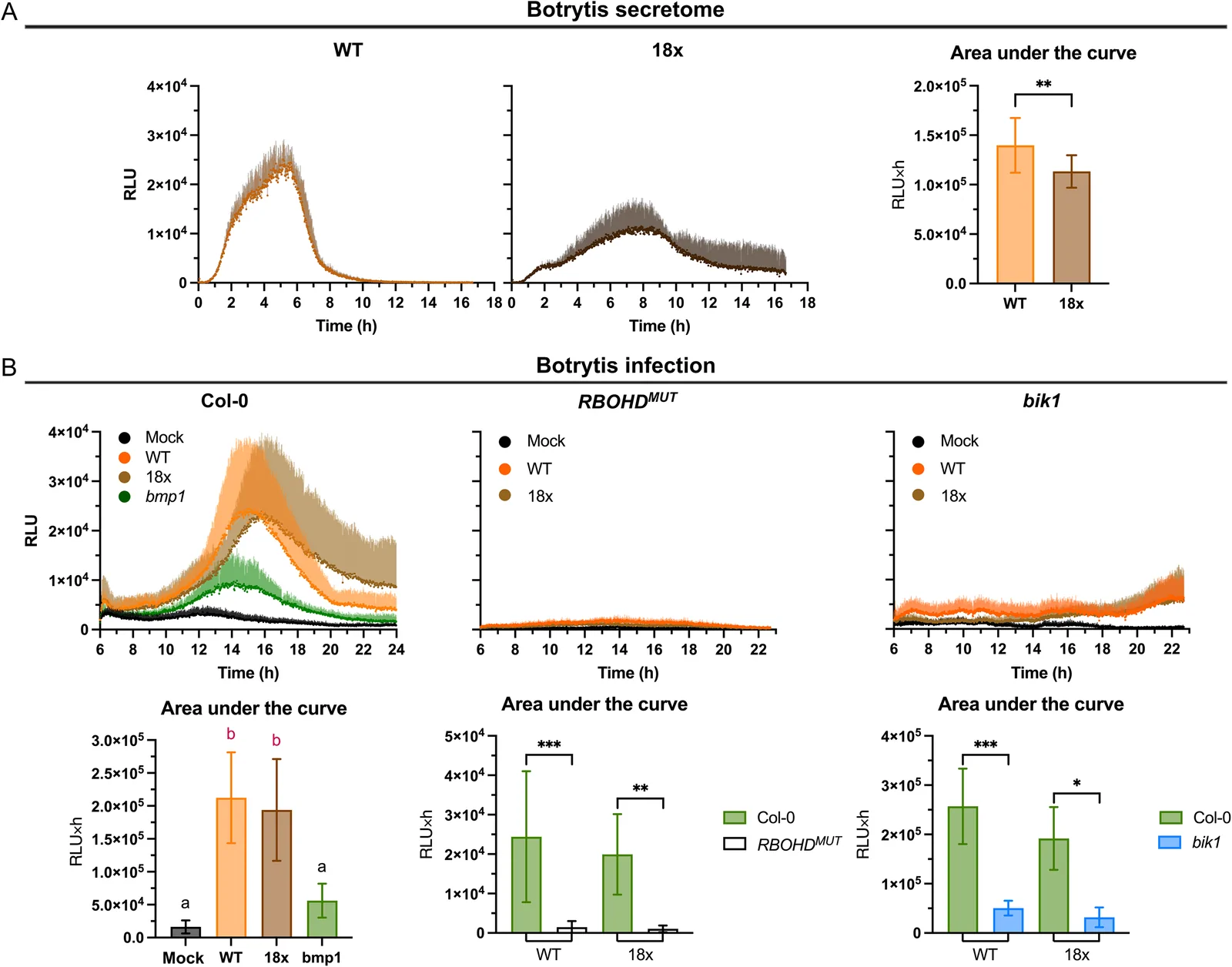
ROS release from Arabidopsis leaf discs triggered by secretome treatment and by *B. cinerea* infection. **(A)** ROS production in Arabidopsis Col-0 induced by the secretomes of *B. cinerea* WT and 18x mutant, and the corresponding area under the curve (AUC; right). **(B)** ROS production induced by infection with pregerminated *B. cinerea* WT, 18x and *bmp1* mutant conidia, or mock treatment, in Arabidopsis Col-0, *RBOHD^MUT^* and *bik1* (upper panels; *bmp1* was tested in Col-0 only), and the corresponding AUC (lower panels). Time-course graphs show one representative experiment, with shaded areas indicating mean ± SD of 3–6 leaf discs (technical replicates). AUC bars in **(B)** represent mean ± SD of 3–6 independent experiments. Statistical significance in **(A)** and in the *RBOHD^MUT^* and *bik1* comparisons with Col-0 in **(B)** was assessed using a ratio paired t-test, *p<0.033, **p<0.002, ***p<0.001. For Col-0 in **(B)**, statistical analysis was performed by one-way ANOVA followed by Tukey’s *post hoc* test (α = 0.05); significant differences are indicated by CLD. Raw data and sample sizes are provided in **Supplementary Table S4**. RLU: relative light units.

Strikingly, ROS production in response to *B. cinerea* infection was largely abolished in the *RBOHD^MUT^* mutant (16-fold lower AUC than in Col-0), with an AUC of 1.5-fold over the mock control compared to 9.2-fold in Col-0 (**Figure 2B**); and strongly reduced in a *bik1* mutant which is unable to activate RBOHD (5.1-fold lower AUC than in Col-0), although the remaining response was still above that of the mock control (**Figure 2B**). These data show that RBOHD is the major source of the ROS production induced by fungal infection.

### *B. cinerea* ROS-detoxification mutants show differentially increased sensitivity to superoxide and H_2_O_2_

By using a CRISPR/Cas9-based transformation protocol (Leisen et al., 2022), we reevaluated the role of several ROS-detoxification systems for growth and infection of *B. cinerea*. For this purpose, we generated marker-free mutants of ROS detoxification-related proteins that have been characterized previously by other groups, namely transcription factor AP1 (Temme and Tudzynski, 2009), superoxide dismutase SOD1 (López-Cruz et al., 2017), catalase CAT2 (Schouten et al., 2002), and cytochrome c-peroxidase CCP1 (Yang et al., 2022). Molecular confirmation of the mutants is shown in **Supplementary Figure S5**. In addition to the single mutants *ap1*, *sod1*, *cat2*, and *ccp1*, an *ap1sod1* double mutant and an *ap1sod1cat2* triple mutant were also generated (**Supplementary Figure S5**). We further constructed a mutant lacking protein kinase Srpk1 which has previously been described to be required for activation of ROS-detoxification enzymes in *Fusarium oxysporum* and *B. cinerea* (Zhang et al., 2023). However, because of the strong growth defect of the obtained *srpk1* mutant (**Supplementary Figure S6**), which has not been described by Zhang et al. (2023), it was excluded from further analysis. All other mutants displayed almost normal germination, growth, sclerotia formation, and sporulation (**Supplementary Figure S7**), indicating no major role of these ROS detoxification systems during unstressed growth and differentiation. In contrast, clear phenotypes of the mutants were observed in the presence of menadione (a source of O_2_^•-^) and H_2_O_2_ (**Figure 3**). Compared to WT, the *sod1* mutant was highly sensitive to menadione, and moderately sensitive to H_2_O_2_ (**Figure 3A**). The growth of the *ccp1* and *cat2* mutants was not affected by menadione, but by H_2_O_2_, and the *ccp1* mutant was more sensitive than *cat2* (**Figure 3A**). The *ap1* mutant was sensitive to menadione and highly sensitive to H_2_O_2_ (**Figure 3A**). All these results were in agreement with previous studies (López-Cruz et al., 2017; Yang et al., 2022; Schouten et al., 2002; Temme and Tudzynski, 2009). The double and triple mutants were similarly sensitive to menadione as the *sod1* mutant (**Figure 3A**), and similarly sensitive to H_2_O_2_ as the *ap1* mutant (**Figure 3A**). All mutants lacking *ap1* failed to germinate under 5 mM H_2_O_2_, while germination of the *ccp1* and *cat2* mutants was more inhibited by H_2_O_2_ than the *sod1* mutant (**Figure 3B**).

**Figure 3:**
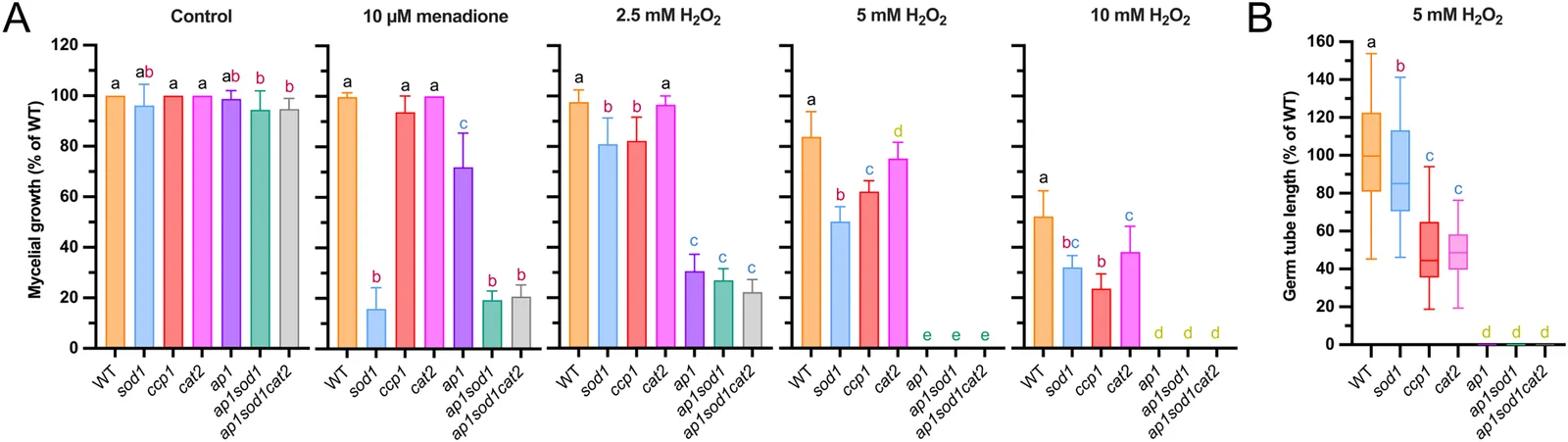
Mycelial growth and sensitivity of *B. cinerea* mutants to menadione and H_2_O_2_. **(A)** Mycelial growth (radial colony diameter, % of WT on control medium) after 6 days of incubation on ME agar supplemented with 10 µM menadione or 2.5, 5 or 10 mM H_2_O_2_ (control: ME without supplement); bars represent mean ± SD. **(B)** Germ tube length (% of WT) after 7.5 h in medium containing 5 mM H_2_O_2_. The experiments were repeated three times, with at least 3 plates per test and strain. Raw data and sample sizes are provided in **Supplementary Table S4**. Statistical analysis was performed by one-way ANOVA followed by Tukey’s *post hoc* test (α = 0.05); significant differences are indicated by Compact Letter Display (CLD).

### The importance of ROS detoxification for infection of *B. cinerea* is dependent on the host tissue

To evaluate the role of fungal ROS detoxifying systems during infection, infection tests with the mutants were performed in simultaneous experiments on leaves of four host species: tomato, *Phaseolus vulgaris* bean, *Nicotiana benthamiana* tobacco and Arabidopsis. Overall, the *sod1* mutant showed the strongest reduction in infection of all single mutants, followed by the *ap1* mutant, which was consistently reduced compared to WT except on Arabidopsis (**Figure 4**). The *ccp1* mutant was weakly reduced or similar to WT, while the *cat2* mutant remained unaffected in infection (**Figure 4**). All mutants showed only small, if any, reductions in virulence on tomato leaves (**Figure 4A**), whereas stronger phenotypes were observed on leaves of bean (**Figure 4B**), tobacco (**Figure 4C**) and Arabidopsis (**Figure 4D**). The phenotypes of *ap1sod1* and *ap1sod1cat2* mutants were similar to those of the *sod1* single mutant, except on Arabidopsis, where the virulence of the *ap1sod1* mutant but unexpectedly not the *ap1sod1cat2* mutant was further reduced. A two-way ANOVA with mutant strain and host as factors showed a significant strain × host interaction (F(18, 1425) = 13.01, P < 0.001), confirming that the effect of the mutations on virulence depends on the host species. These results support the role of ROS detoxification for successful infection of *B. cinerea*, and a major role of SOD1 and AP1, but no role for CAT2 and only a minor role for CCP1. Furthermore, the importance of fungal ROS-detoxification is host-dependent, suggesting that the size of the ROS burst and its role in pathogen defense differ among plant tissues.

**Figure 4:**
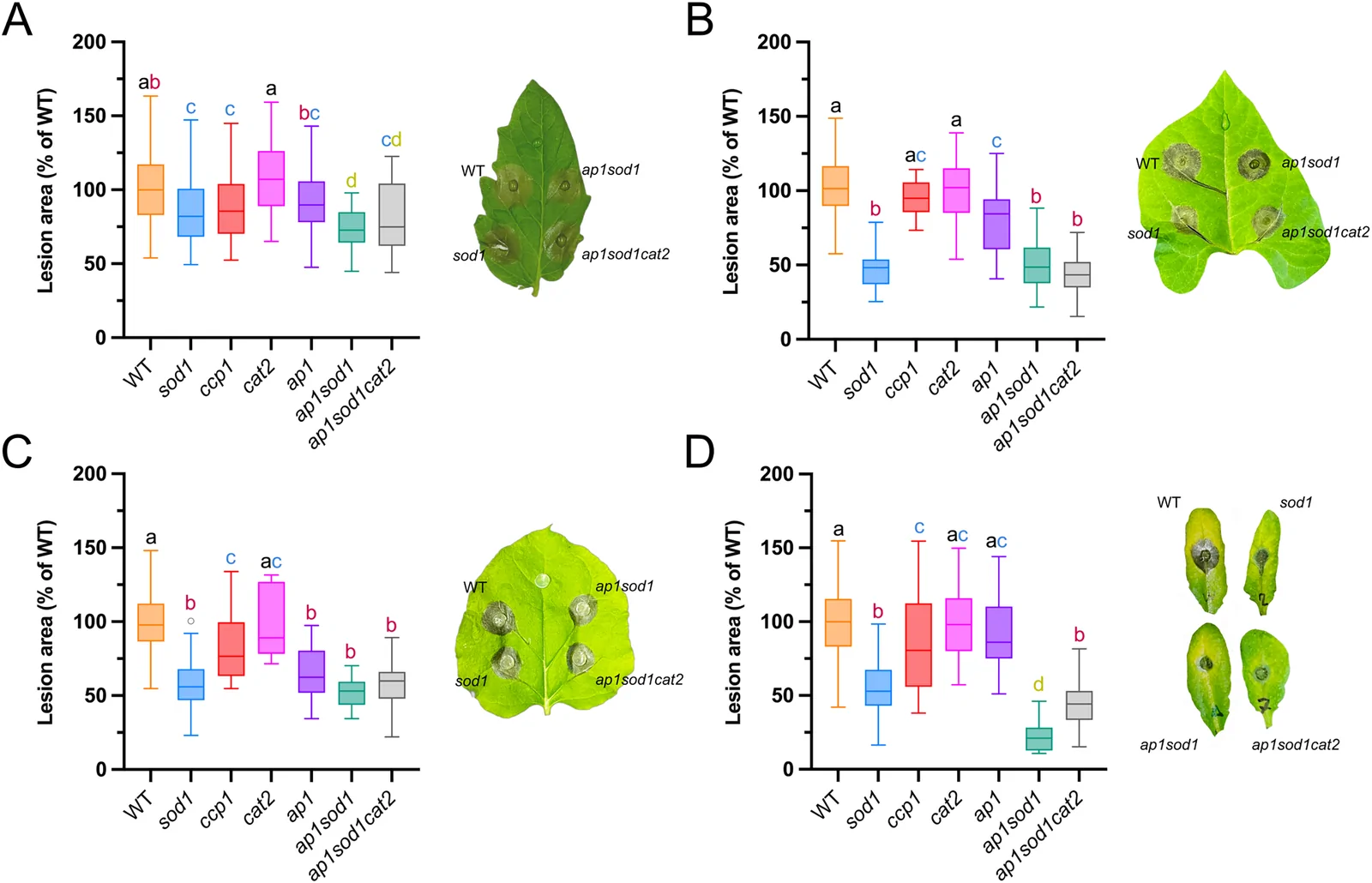
Infection behavior of *B. cinerea* ROS detoxification mutants on leaves of different host plants. **(A)** Detached tomato leaves; **(B)** attached *Phaseolus vulgaris* bean leaves; **(C)** attached *Nicotiana benthamiana* leaves; **(D)** attached Arabidopsis Col-0 leaves (all 60 hpi); lesion areas are expressed as % of WT. The infections were conducted three times, with five plants per host and at least three infections per plant and strain in each replicate. Raw data and sample sizes are provided in **Supplementary Table S4**. Within each host, strains were compared by one-way ANOVA followed by Tukey’s *post hoc* test (α = 0.05); significant differences are indicated by CLD. To test whether the effect of the mutations depended on the host, a two-way ANOVA with strain, host and their interaction as factors was performed (see Results section).

### Fungal superoxide dismutase SOD1 increases H_2_O_2_ concentrations during the oxidative burst

Based on the observation that several *B. cinerea* detoxification proteins contribute to infection, we examined whether they also influence apoplastic H_2_O_2_ levels during the ROS burst. Using the luminol-based assay established in this study, pregerminated detoxification mutants induced ROS responses similar to WT (**Figure 5A**), with the *sod1* mutant showing a tendency towards a lower response (AUC 0.65-fold of WT) (**Figure 5A**). An explanation for this observation could be that the absence of SOD1 activity in the apoplast leads to reduced conversion of superoxide to H_2_O_2_. Unconventional secretion of SOD1, which does not contain a classical signal peptide, is well documented in yeast and filamentous fungi (Tian et al., 2021), and apoplastic SOD1 has also been found in *B. cinerea* (Zhu et al., 2017; Klug et al., 2024). Interestingly, while the *ap1sod1* mutant induced a similarly reduced ROS burst as the *sod1* mutant, the ROS burst induced by the *ap1sod1cat2* triple mutant was similar to that induced by the WT (**Figure 5A**). An explanation of this result could be that the loss of a major apoplastic catalase leads to a slower breakdown of H_2_O_2_, thereby compensating for the simultaneous loss of SOD1.

**Figure 5:**
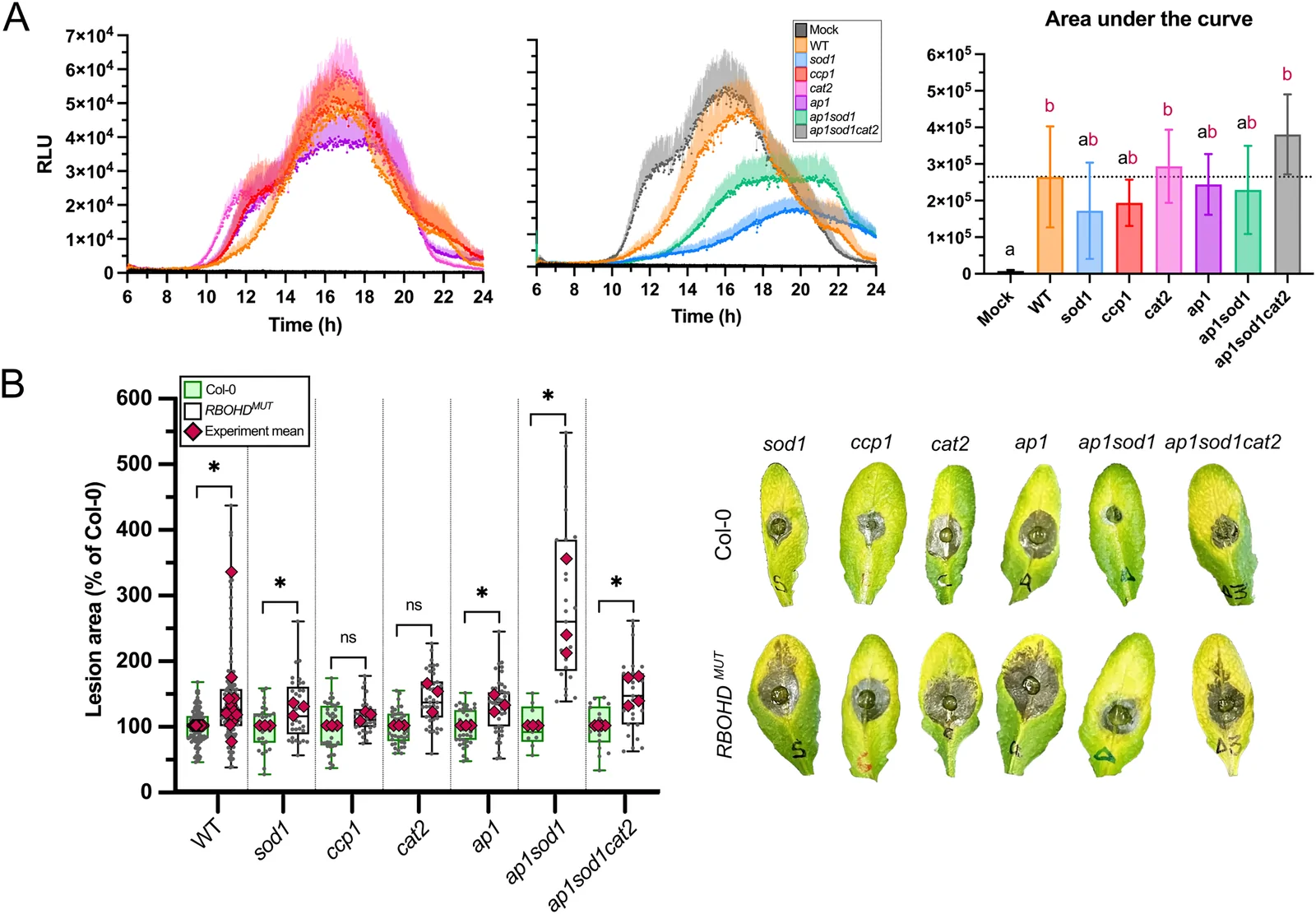
Infection and ROS production induced by *B. cinerea* ROS-detoxification mutants in Arabidopsis Col-0 and *RBOHD^MUT^ leaves.* **(A)** ROS release from Col-0 leaf discs triggered by pregerminated *B. cinerea* WT and ROS-detoxification mutants, or mock treatment. Left: *ccp1*, *cat2* and *ap1*. Center: *sod1*, *ap1sod1* and *ap1sod1cat2*. In both panels, *B. cinerea* WT (orange trace) is shown as a reference. Traces show one representative experiment, with shaded areas indicating mean ± SD of 3–6 leaf discs. Right: area under the curve (AUC, 6–22.5 hpi) across independent experiments, with bars representing the mean ± SD of independent experiments (n = 4; *ap1sod1cat2*, n = 3). The dotted line indicates the mean of WT. Statistical analysis was performed by one-way ANOVA followed by Tukey’s *post hoc* test (α = 0.05); significant differences are indicated by CLD. **(B)** Left, box-and-whisker plots showing lesion sizes in Col-0 (green) and *RBOHD^MUT^* (white) leaves 48 hpi with *B. cinerea* WT and ROS-detoxification mutants. Grey dots show individual lesions; red diamonds show the mean of each independent experiment. Assays were conducted at least three times (WT, 13 independent experiments; *ap1sod1cat2*, 4; other mutants, 3), with six plants per line and at least two infections per plant and condition in each replicate. Raw data and sample sizes are provided in **Supplementary Table S4**. Statistical significance was assessed on the experiment means: for each experiment, the ratio of the mean lesion size on RBOHD^MUT^ to that on Col-0 was calculated, and the log-transformed ratios were compared to zero with a one-sample t-test. *p<0.05; ns, not significant. Right, images of *B. cinerea*-infected Arabidopsis leaves (48 hpi).

When the mutants were inoculated on leaves of Arabidopsis *RBOHD^MUT^*, significantly larger lesions were formed compared to Col-0 for all strains except the *ccp1* and *cat2* mutants, similar to what was already shown for *B. cinerea* WT (**Figure 5B**). For the *ap1sod1* double and the *ap1sod1cat2* triple mutants, this increase was stronger compared to the increase observed for *B. cinerea* WT on *RBOHD^MUT^*: They were strongly reduced in virulence on Col-0, and in many cases did not form any lesions, but more frequently showed lesions on the mutant (31% and 59% in Col-0 for *ap1sod1* and *ap1sod1cat2*, respectively, versus 67% and 93% in *RBOHD^MUT^*). These data indicate that the loss of the plant oxidative burst partially offsets the impaired ROS detoxification ability, which further highlights the antifungal effect of the ROS burst against *B. cinerea*.

### *B. cinerea* secretome induces faster cytoplasmic oxidation in Arabidopsis *rbohD* mutants

To assess the susceptibility of the *rbohD* mutant to the phytotoxic secretome, an assay was performed using a redox sensor based on the glutaredoxin 1 (GRX1) fused with GFP (Gutscher et al., 2008). Leaf discs of Col-0 Grx1-roGFP2 and *rbohD* Grx1-roGFP2 were treated with 2 µg/mL of the *B. cinerea* WT secretome, and the kinetics of cytoplasmic oxidation was monitored as an indicator of loss of cellular redox homeostasis. The oxidation state of the secretome-treated Col-0 Grx1-roGFP2 leaf discs was elevated compared to untreated controls but increased only slowly during the measurement period. In contrast, the oxidation state of the *rbohD* mutant continuously increased and reached full oxidation already after 11 h (**Figure 6A**). At later time points, apparent OxD values in the *rbohD* line exceeded the theoretical maximum of 1.0. This is most likely an artifact of the progressive collapse of the secretome-treated leaf discs, which raises tissue autofluorescence in the 405 nm excitation channel and thereby inflates the calculated OxD. The faster oxidation kinetics of the *rbohD* line relative to Col-0 are, however, unaffected by this artifact. These results indicate that the RBOHD-generated oxidative burst contributes to the maintenance of cellular redox homeostasis during phytotoxic stress, by preserving a reduced cytosolic glutathione redox potential.

**Figure 6:**
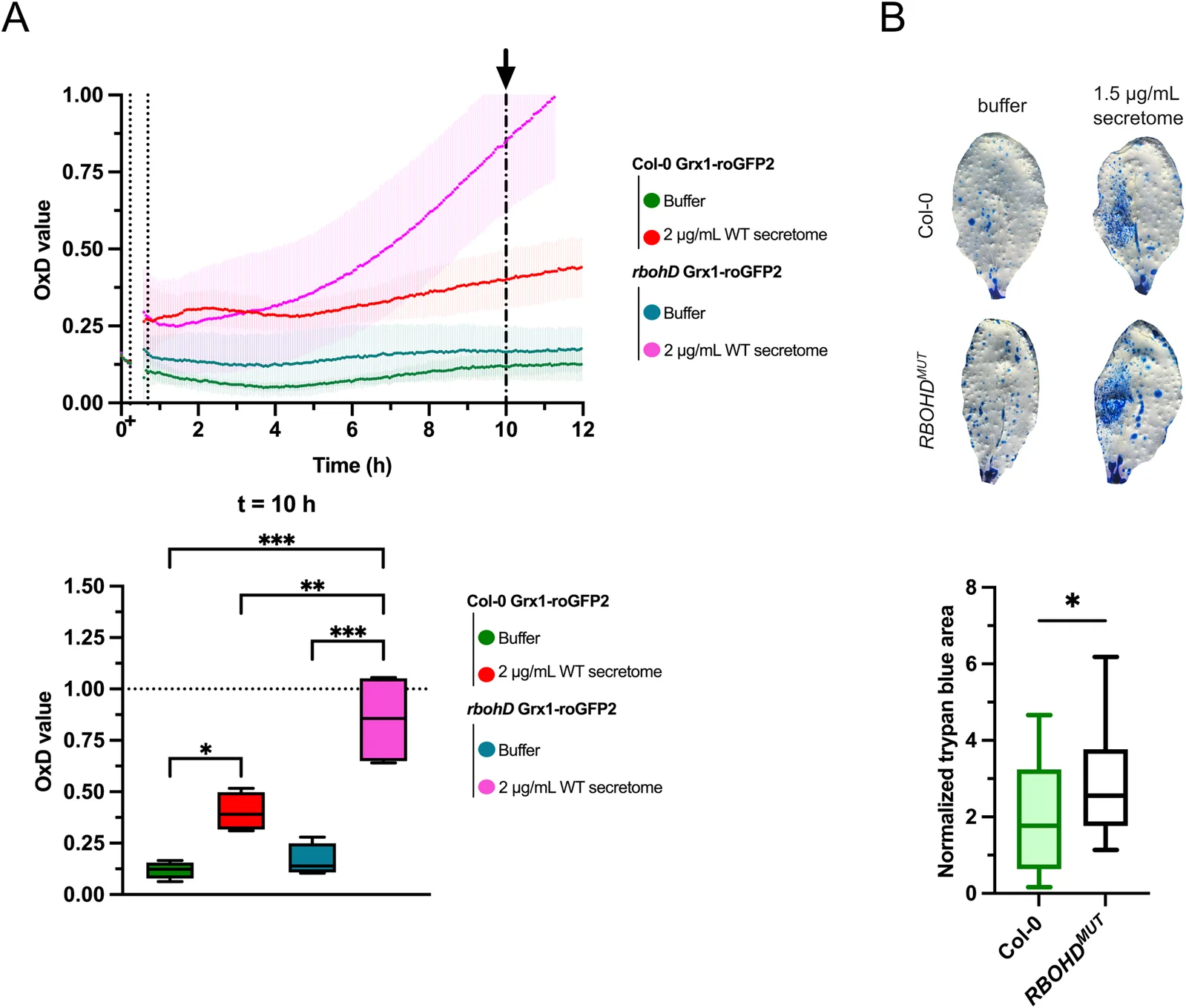
Effects of *B. cinerea* secretome on the cytosolic redox potential and cell death of Arabidopsis. **(A)** Leaf discs of Col-0 Grx1-roGFP2 and *rbohD* Grx1-roGFP2 were treated with buffer (control) or 2 µg/mL *B. cinerea* WT secretome. Top, time-course measurement of the degree of oxidation (OxD) determined by ratiometric fluorescence; lines and shaded areas show mean ± SD. Two dotted lines indicate opening of the plate for addition of DTT, H_2_O_2_ or secretome and start of measurement. Bottom, box-and-whisker plot from OxD values at t = 10 h (arrow on top graph), used for statistical comparison between genotypes and treatments; the dotted line indicates the theoretical maximum OxD of 1.0. Raw data and sample sizes are provided in **Supplementary Table S4**; the time-course graph is one representative of three independent experiments. Statistical analysis was performed by one-way ANOVA followed by Tukey’s *post hoc* test, *p<0.05, **p<0.01, ***p<0.001. **(B)** top, representative images of Trypan Blue-stained Arabidopsis leaves 24 h after infiltration with buffer or 1.5 µg/mL *B. cinerea* WT secretome; bottom, quantification of the stained area, expressed as normalized Trypan Blue-stained area. Raw data and sample sizes are provided in **Supplementary Table S4**. Statistical significance was assessed using an unpaired t-test, *p<0.05.

To confirm that the observed effects were specifically related to RBOHD activity, the *RBOHD^MUT^* mutant was analyzed. As no redox sensor line is available in the background of this mutant, cell death was assessed 24 h after infiltration with *B. cinerea* WT secretome. Both Col-0 and *RBOHD^MUT^* leaves showed increased Trypan Blue staining compared to mock-infiltrated controls, but quantification of the stained areas showed weakly increased cell death in the *RBOHD^MUT^* mutant compared to Col-0 (**Figure 6B**). These results suggest that RBOHD activity protects plant cells from excessive oxidative or other phytotoxic stress.

### Arabidopsis Col-0 and RBOHD^MUT^ leaves exhibit a similar transcriptional response to *B. cinerea* infection

RBOHD is considered a key component of the PTI response, and it is assumed that in addition to its antimicrobial activity, the signaling role of RBOHD-generated H_2_O_2_ is important for the immune response (Alvarez et al., 1998). To investigate if the RBOHD-triggered ROS burst is involved in the transcriptional response to *B. cinerea* infection, a comparative RNA-seq analysis of Arabidopsis Col-0 and *RBOHD^MUT^* leaves infected with *B. cinerea* was performed, at 48 hpi when secondary lesions started to develop. Compared to mock treatment, *B. cinerea* infection led to differential expression (FDR ≤ 0.05, |FC| ≥ 1.5) of 14,049 genes in Col-0 and 14,120 genes in *RBOHD^MUT^*, including strong induction of multiple genes related to salicylic acid (SA) and jasmonic acid/ethylene (JA/ET) signaling, phytoalexin biosynthesis, and stress responses (**Figure 7A**; **Supplementary Table S4**), in agreement with previously described Arabidopsis transcriptional responses to *B. cinerea* infection (Wei et al., 2024). Unexpectedly, the gene expression profiles of the two Arabidopsis lines were highly similar (**Figure 7**). Of these DEGs, 13,180 were shared (93.8% of the Col-0 and 93.3% of the RBOHD^MUT^ DEGs), 99.97% of the shared DEGs were regulated in the same direction, and the infection-induced log_2_ fold changes of the two genotypes were strongly correlated (**Figure 7A**; **Supplementary Figures S9B, C**). Only 15 of 27,655 annotated genes (0.05%) were differentially expressed between Col-0 and *RBOHD^MUT^* leaves after infection (**Figure 7A**; **Supplementary Figure S9D**; **Supplementary Table S2**). In addition, gene set enrichment analysis (GSEA) of the genotype comparison did not reveal any significantly enriched defense-, hormone-, ROS/redox- or cell death-related GO term (lowest FDR = 0.24); only flowering-related terms (driven by MAF4 and MAF5), plastid organization and amino acid transport reached FDR < 0.05 (**Supplementary Figure S9E**). For genes associated with the glutathione/ascorbate and thioredoxin pathways, similar infection-induced expression in Col-0 and *RBOHD^MUT^* plants was observed (**Supplementary Table S3**). Thus, transcriptional adjustment of cytosolic antioxidant capacity seems to occur largely independent of RBOHD activity at 48 hpi. To further confirm the highly similar gene expression in these two lines, the infection-induced expression of selected genes with known functions in JA/ET-, SA-, ROS-, camalexin- and redox-related pathways are shown (**Figure 7B**).

**Figure 7:**
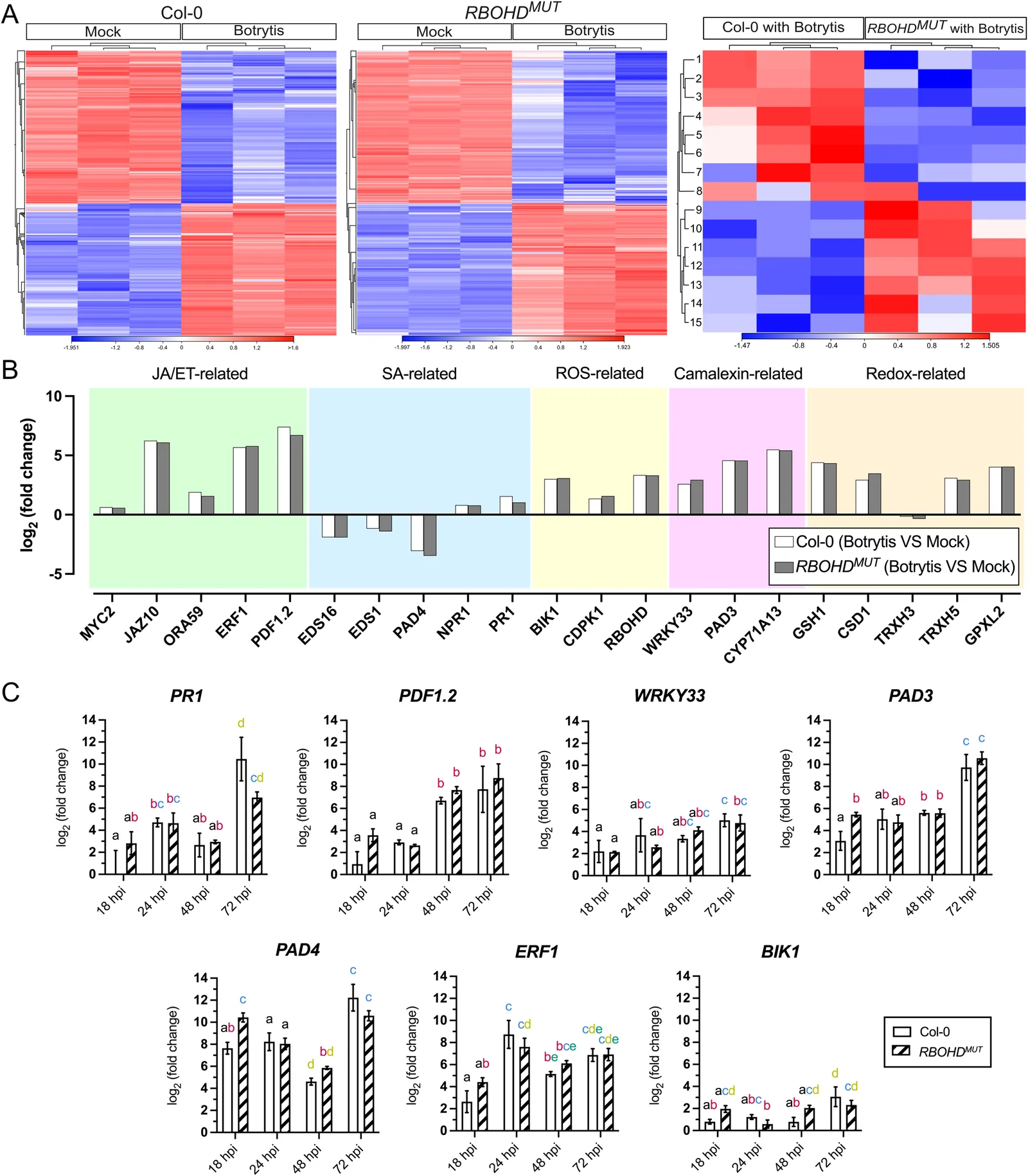
Gene expression profiles of Arabidopsis Col-0 and *RBOHD^MUT^* after *B. cinerea* infection. **(A)** Heat maps showing genes up- and downregulated in mock- and *B. cinerea*-infected Col-0 (left) and *RBOHD^MUT^* (center) leaves (48 hpi; 3 biological replicates per genotype and treatment; FDR ≤ 0.05, |FC| ≥ 1.5); differentially expressed genes between Col-0 and *RBOHD^MUT^* are shown in the right heat-map, with the gene names listed in **Supplementary Table S2**. **(B)** *B. cinerea*-induced changes in expression (log_2_ fold change, infected vs mock; RNA-seq, 48 hpi) of selected JA/ET-, SA-, ROS-, camalexin- and redox-related genes. **(C)** Time course of defense marker gene expression in Col-0 and *RBOHD^MUT^* after *B. cinerea* infection. Gene expression values are shown as log_2_ fold changes relative to mock-treated plants, with bars representing the mean ± SD. Raw data and sample sizes are provided in **Supplementary Table S4**. Statistical analysis was performed by two-way ANOVA followed by Šidák’s multiple comparisons test (α = 0.05); significant differences are indicated by CLD.

To compare the time course of expression of defense-related genes in WT and *RBOHD^MUT^* plants from early to late stages of infection, RT-qPCR analysis was performed at 18, 24, 48 and 72 hpi with representative marker genes for SA-dependent responses (*PR1*, *PAD4*; Uknes et al., 1992; Jirage et al., 1999), JA- and ethylene-dependent responses (*PDF1.2*, *ERF1*; Penninckx et al., 1996; Lorenzo et al., 2003), for transcription factors involved in defense gene activation and camalexin biosynthesis (*WRKY33*, *PAD3*; Zheng et al., 2006; Mao et al., 2011), and for the PTI regulator BIK1 (Veronese et al., 2006). Again, the expression profiles of the two Arabidopsis lines were very similar, and they did not indicate a delayed transcriptional response of the activation-impaired RBOHD mutant, with the exception of PAD3 and PAD4, which showed significantly higher induction in RBOHD^MUT^ than in Col-0 at 18 hpi (**Figure 78C**). RT-qPCR analysis of *PR1*, *PDF1.2* and *WRKY33* also did not reveal major differences in their temporal expression patterns between the *rbohD* mutant and WT plants (**Supplementary Figure S8**).

## Discussion

The oxidative burst is a universal defense mechanism of plants challenged with a variety of pathogens (Torres, 2010). In the case of infection with *B. cinerea*, however, the situation is complicated by the fact that this necrotroph induces HR as part of its infection strategy. Apoplastic ROS have long been proposed to promote HR (Levine et al., 1994; Govrin and Levine, 2000), and this view is still maintained in recent work and reviews (Cai et al., 2020; Mengiste and Liao, 2025). Our data challenge this view and instead support a protective function of the apoplastic oxidative burst during infection by *B. cinerea*. The loss of RBOHD activity, either in *rbohD* null mutants or in activation-impaired *RBOHD^MUT^* plants, slightly but consistently increased susceptibility. These results suggest that the apoplastic ROS can act as a barrier that limits fungal colonization rather than promoting cell death. The high ROS sensitivity of the ungerminated conidia and their increased infection of RBOHD-defective plant mutants support the idea that the oxidative burst inhibits primarily their germination. Overall, the protective role of the RBOHD-dependent ROS burst against *B. cinerea* infection is rather weak, and therefore might not have been detected by Galletti et al. (2008). On the other hand, the studies with the tomato *sitiens* mutant indicate that the ROS burst can be strong and inhibitory for *B. cinerea* infection (Asselbergh et al., 2007).

To clarify how the ROS burst is triggered by *B. cinerea* and how it is generated, we adapted a luminol-peroxidase-based ROS (H_2_O_2_) detection assay for use with *Botrytis*-infected leaf discs which allows the dynamics of extracellular ROS production to be monitored during infection. Previous studies have analyzed the temporal accumulation of ROS in plant cell suspensions or leaves in response to *B. cinerea* using biochemical and *in vivo* luminol-based approaches (Unger et al., 2005; Magnin-Robert et al., 2015). In the study by Magnin-Robert et al. (2015), leaf discs were placed in luminol/HRP and germinated conidia were added to the assay solution. With this system, a transient ROS response was observed, starting at 2.5 h and peaking at about 6 h. However, in contrast to our approach, no fungal infection was monitored in this study. Other studies have used staining of Arabidopsis leaves with diaminobenzidine (DAB) or fluorescent probes to visualize H_2_O_2_ accumulation. These classical methods only provide endpoint measurements, and do not allow discrimination between H_2_O_2_ generated as part of the oxidative burst and artifacts coming from the infected and dead tissue. In contrast, our assay offers a quantitative *in vivo* analysis of the ROS dynamics. However, it is limited by the fact that measurements can be started only 6 h after inoculation of the leaf discs, in order to allow conidia attachment and germination before flipping the leaf discs, which precludes the detection of early ROS responses. In our study, H_2_O_2_ accumulation was not detected until 10 h after inoculation, although we cannot exclude a transient, PTI-like ROS production during the first 6 h. Our data, together with the findings for the penetration-deficient *bmp1* mutant, indicate that the main ROS burst induced by *B. cinerea* occurs during the penetration phase.

*B. cinerea* secretes a broad array of phytotoxic metabolites, CDIPs and cell wall–degrading enzymes during infection. In our assays with leaf discs, purified secretomes induced ROS production in a toxicity-dependent manner within one hour after their addition. In contrast, when the leaf discs were inoculated with fungal conidia, infection-induced ROS accumulation did not start until about 10 h after inoculation. These data show that treatments with phytotoxic secretomes are very different from fungal infection, and cannot easily be compared. Whereas the ROS produced was similar in response to WT and 18x mutant infection, the penetration-deficient MAP kinase *bmp1* mutant induced a much smaller ROS response. This suggests that perception of invasive growth by the host is required to initiate sustained ROS production, whereas the toxic secretome seems less important for triggering the ROS burst during the early stages of infection.

Inactivation of RBOHD abolished extracellular ROS accumulation during infection almost completely, which indicates that RBOHD is indispensable for the ROS response to *B. cinerea* infection. These results are partly in agreement with previous studies which used the DAB staining method. For example, the ROS bursts triggered by incompatible strains of *P. syringae* and the oomycete *Peronospora parasitica* were found to be dependent on RBOHD and, to a lesser extent, RBOHF (Torres et al., 2002), and in the interaction with the necrotroph *Alternaria brassicicola*, RBOHD, together with two peroxidases Prx33 and Prx34, was found to be responsible for the oxidative burst (Kámán-Tóth et al., 2019). In *B. cinerea*-inoculated leaf discs of a *bik1* mutant, we observed a strong reduction of the ROS burst, consistent with the critical role of BIK1 in PTI-dependent activation of RBOHD (Kadota et al., 2014).

For successful infection, *B. cinerea* requires efficient detoxification of host-derived ROS, and mutants impaired in ROS-detoxification showed reduced virulence, with SOD1 and the transcription factor AP1 being particularly important. The reduced virulence of *sod1* mutants confirms previous work (Rolke et al., 2004; López-Cruz et al., 2017), while the *ap1* mutant has been reported to be unaffected in virulence (Temme and Tudzynski, 2009). This discrepancy might reflect differences in the evaluation of the infection tests. The importance of SOD1 and AP1 is consistent with observations in other pathogens. Deletion of SOD1 in *Verticillium dahliae* and *Fusarium graminearum* led to reduced virulence (Tian et al., 2021; Yao et al., 2016), and the loss of AP1 similarly impaired infection in *Magnaporthe oryzae*, *Colletotrichum gloeosporioides*, and *Ustilago maydis* (Guo et al., 2011; Sun et al., 2016; Molina and Kahmann, 2007). CAT2 was confirmed to be not essential (Schouten et al., 2002), probably due to the redundancy of H_2_O_2_-degrading enzymes in the secretome. The *ccp1* mutant showed no or only a mild phenotype, in contrast to the results of Yang et al. (2022). Although they presented evidence that CCP1, in addition to its mitochondrial localization, is also secreted, it has never been detected in published secretome data of *B. cinerea* (e.g., Müller et al., 2018; Leisen et al., 2022), suggesting a minor role of CCP1 in extracellular H_2_O_2_ detoxification. In addition to the proteins investigated in this study, other redox-related proteins have been shown to contribute to ROS detoxification and virulence (Viefhues et al., 2014; Zhang et al., 2025). Because the genes for some of these proteins are positively regulated by AP1 in *B. cinerea* and other fungi, it makes sense that AP1 contributes to ROS tolerance during infection (Temme and Tudzynski, 2009; Simaan et al., 2019). Together, it is evident that *B. cinerea* uses diverse mechanisms that allow it to maintain redox equilibrium when exposed to oxidative stress in the environment or during infection. The variable lesion phenotypes of ROS-detoxification mutants across different host species suggest that the intensity and defensive relevance of the oxidative burst may differ between plants. For example, the smaller differences in lesion sizes between WT and ROS-detoxification mutants on tomato leaves could indicate that the *B. cinerea*-induced ROS burst in tomato leaves is weaker than that in the other leaf tissues. The multitude of fungal ROS detoxification systems, beyond those investigated in this study, illustrates that they have evolved to be sufficiently robust to cope with different oxidative environments.

The tendency towards reduced accumulation of H_2_O_2_ during infection with *sod1* mutants may indicate that a fungal detoxification enzyme can modulate the composition of host-derived ROS after RBOHD activation. RBOHD-derived superoxide is normally converted to H_2_O_2_ by BcSOD1; in the absence of this enzyme, superoxide may persist locally, resulting in reduced accumulation of H_2_O_2_ despite intact RBOHD activity. This suggests an important local role for RBOHD-generated superoxide that is counteracted by secreted fungal SOD1. The partial restoration of H_2_O_2_ levels in *ap1sod1* double mutants, and almost complete restoration in mutants additionally lacking catalase activity, further support the idea that fungal detoxification pathways may dynamically modulate extracellular ROS. The role of extracellular SOD1 in detoxifying O_2_^•-^ to H_2_O_2_ has been demonstrated in several pathogens *in vitro* (Schatzman and Culotta, 2018), but *in vivo* measurements of host-derived ROS have not been reported for other pathogens.

In addition to its extracellular antifungal activity, RBOHD activity contributed to the maintenance of intracellular redox balance under phytotoxic stress. In the rbohD knockout mutant, cytosolic oxidation progressed much more rapidly, whereas cell death was only modestly increased in the activation-impaired *RBOHD^MUT^* line compared to Col-0. This difference in the severity of the phenotypes might be explained by the complete loss of RBOHD in the *rbohD* knockout line, whereas the line expressing RBOHD with S39A/S339A/S343A exchanges still has some enzymatic activity (Berlanga et al., 2024), which might be sufficient to maintain some of its functions related to redox homeostasis. We have previously shown that infiltration of the phytotoxic *B. cinerea* secretome into barley leaves expressing the Grx1-roGFP2 sensor resulted in oxidation of the cytoplasm, which might incapacitate proteins required for effective defense responses (Bohle et al., 2024). In the present measurements, apparent OxD values close to and above the theoretical maximum of 1.0 in secretome-treated *rbohD* leaf discs are best explained by increased autofluorescence caused by tissue collapse rather than by a genuine redox signal, particularly in the 405 nm excitation range that serves as the oxidized channel of roGFP2, which inflates the calculated oxidation degree (Rosenwasser et al., 2010). To compare genotypes, OxD values were quantified at t = 10 h. At this time point, secretome-treated *rbohD* Grx1-roGFP2 leaf discs showed significantly higher oxidation than both secretome-treated Col-0 leaf discs and mock-treated controls of either genotype (**Figure 6A**). Together, these results suggest that RBOHD contributes to the defense against *B. cinerea* by maintaining intracellular redox homeostasis. Previous studies indicate that RBOHD-derived ROS can promote general stress protection. RBOHD has been shown to suppress spreading cell death after infection with necrotrophic pathogens (Torres et al., 2005; Pogány et al., 2009), while RBOHD activity contributes to acclimation to high-light and endoplasmic-reticulum stress (Angelos and Brandizzi, 2018; Zandalinas et al., 2019). The accelerated Grx1-roGFP2 oxidation observed in *rbohD* reports an increasing glutathione redox potential in response to secretome treatment that reaches the upper measurement range of roGFP2 within about 11 hours. This progressive thiol oxidation could be the consequence of various processes that lead to GSH oxidation or removal and/or block GSSG reduction. Thus, in response to the cytotoxic *B. cinerea* secretome, RBOHD seems to support cytosolic redox balance directly or indirectly, e.g. by triggering protective processes and restricting excessive intracellular oxidation. Whether or not this is also relevant during fungal infection remains to be investigated.

RNA-seq profiling showed a similarly strong induction of cytosolic and mitochondrial redox buffering and ROS detoxifying systems in both Col-0 and *RBOHD^MUT^* plants following *B. cinerea* infection. These included thioredoxins (e.g., *TRXh5*, *TRXh8*), glutaredoxin (*GRXC2*), glutathione reductase (*GR1*), as well as ascorbate-dependent enzymes such as *APX1* and *MDAR2* (**Supplementary Table S3**). Despite this compensatory upregulation of redox networks, RBOHD-deficient plants still exhibited accelerated cytosolic oxidation and enhanced cell death, suggesting that transcriptional reinforcement alone is insufficient to restore redox homeostasis during necrotrophic stress. Previous studies have also shown that cytosolic protein redox changes detected by redox biosensors are independent of RBOHD activity (Arnaud et al., 2023).

RNA-sequencing revealed that defense-related signaling pathways were similarly activated in Arabidopsis plants with full or strongly impaired RBOHD activity. Among the small number of downregulated genes in the *RBOHD^MUT^* mutant, At4g36430 encodes a class III cell-wall-localized peroxidase, structurally related to the peroxidases PRX33/34 which have been shown to contribute to the oxidative burst during PTI (Daudi et al., 2012). Plant class III peroxidases form large gene families and can exhibit different catalytic cycles involving ROS and radical formation, which can contribute either to cell wall loosening or to cross-linking of extracellular polymers (Chen and Schopfer, 1999; Francoz et al., 2015). They fulfill a multitude of functions in stress resistance as well as cell wall formation and loosening (Francoz et al., 2015), that cannot be mapped to clades by phylogenetic analysis (Aparato et al., 2024). The function of the peroxidase downregulated in *RBOHD^MUT^*, At4g36430 (AtPRX49), is currently not characterized, but may be interesting to investigate in relation to pathogen responses, especially as its expression has been reported to be induced by bacterial PAMPs (Thilmony et al., 2006).

Because the RNA-seq analysis was performed at 48 hpi, we cannot exclude earlier RBOHD-dependent transcriptional changes, but a time course expression analysis of several defense-related genes from 18 to 72 hpi did not provide evidence for this. PTI-dependent transcriptional reprogramming is primarily mediated by mitogen-activated protein kinases, which can activate defense gene expression independently of RBOHD (Mengiste and Liao, 2025), consistent with the lack of transcriptional differences observed between Col-0 and *RBOHD^MUT^*. In addition, recent work has shown that intracellular ROS, particularly chloroplast-derived redox changes, can act as potent immune signals and shape spatial transcriptional responses around HR lesions independently of apoplastic NADPH oxidase activity (Lukan et al., 2023).

Together, our data support a limited but significant defense-promoting role of the RBOHD-dependent oxidative burst against *B. cinerea* (**Figure 8**). The apoplastic ROS burst represents the earliest induced barrier of the plant that *B. cinerea* must overcome through multiple and redundant ROS-detoxification mechanisms. Although the difference in susceptibility of Col-0 and RBOHD^MUT^ plants to infection was small, it could play a significant role for defense in natural environments, when infections are initiated by single conidia under suboptimal conditions. Furthermore, the small remaining enzymatic activity that has been described for RBOHD^MUT^ plants (Berlanga et al., 2024) might be responsible for their relatively mild phenotype. In addition to its direct antifungal activity, our data suggest that RBOHD also contributes to the maintenance of intracellular redox homeostasis during infection, slowing down cytosolic oxidation and cell death triggered by the fungal secretome.

**Figure 8:**
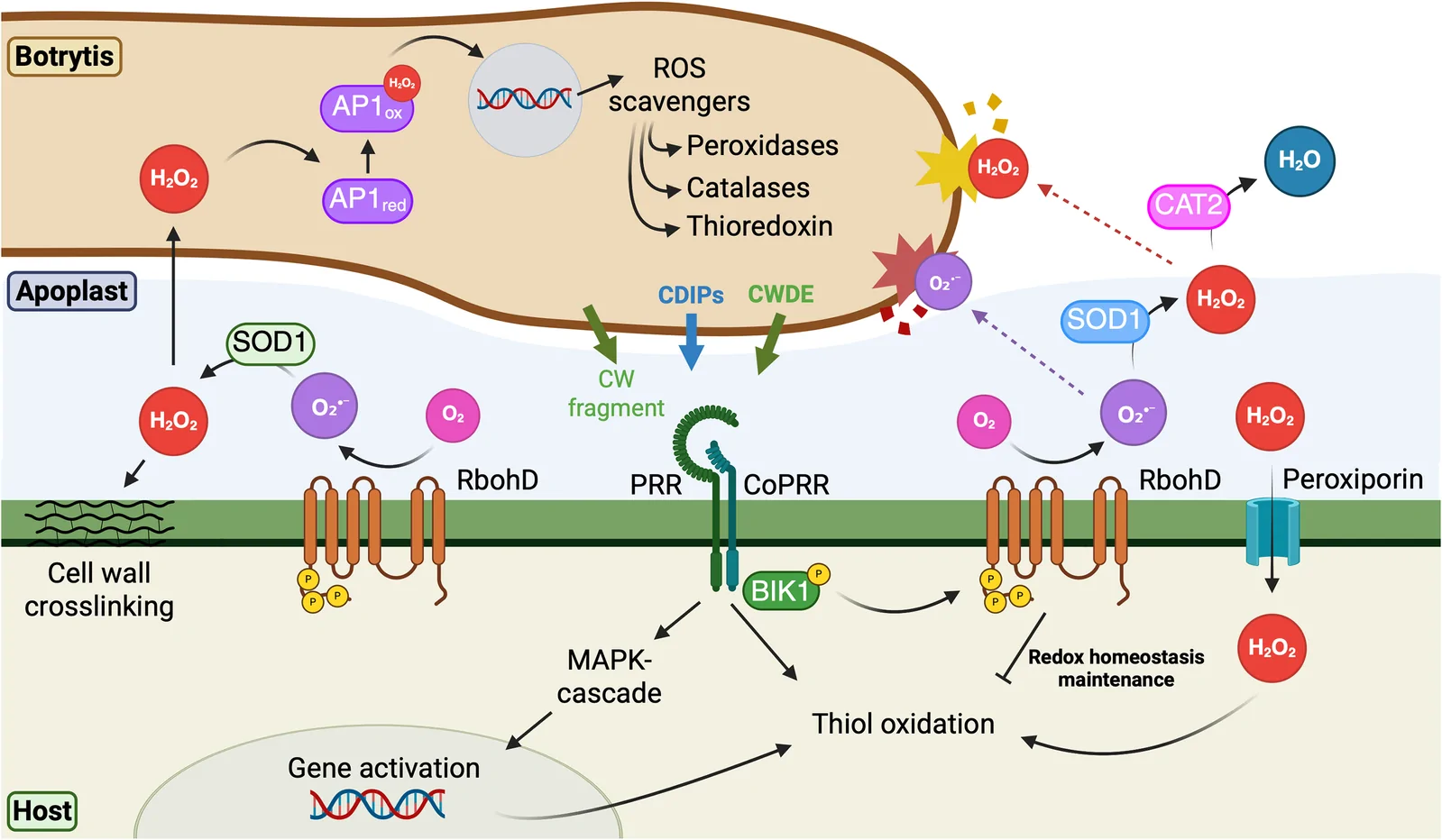
Dynamics of RBOHD-generated ROS during the Arabidopsis–*B. cinerea* interaction. Pathogen or damage perception by pattern-recognition receptors (PRR) and their coreceptors (CoPRR) activates RBOHD via Botrytis-induced kinase 1 (BIK1), generating apoplastic O_2_^•-^ that restricts fungal growth and promotes cell-wall reinforcement. *B. cinerea* mitigates this oxidative stress through multiple ROS-detoxification mechanisms (AP1-regulated ROS-detoxification pathways, SOD1-dependent conversion of O_2_^•-^, H_2_O_2_ scavenging by catalases/peroxidases etc.). In addition, RBOHD activity supports intracellular redox homeostasis under phytotoxic stress, limiting cytosolic oxidation, independently of defense gene activation. CWDE, cell wall-degrading enzymes; CDIPs, cell death-inducing proteins. Partially created with BioRender (https://www.biorender.com/).

Nevertheless, our study did not address the connection between extracellular and intracellular ROS production. To study the contribution of these intracellular ROS sources, the use of genetically encoded H_2_O_2_ biosensors targeted to individual organelles is required (Arnaud et al., 2023). This approach would enable real-time monitoring of compartment-specific ROS signals and help clarify the extracellular-intracellular ROS dynamics during necrotrophic infection.

## Data Availability

The data that support the findings of this study are integrated in the main article, and raw data are provided in **Supplementary Table S4**. The RNA-sequencing data generated in this study have been deposited in the NCBI Gene Expression Omnibus (GEO) and are accessible through GEO Series accession number GSE317547.

## Footnotes

1
https://imagej.nih.gov/ij/

## Supplementary material

Supplementary Figure 1 Effect of ROS-modulating compounds on *B. cinerea* infection in *A. thaliana*. Lesion sizes (*Botrytis* infection area) in Col-0 leaves 48 h after inoculation with WT conidia, following pretreatment (1 h before inoculation) with H_2_O_2_ (100, 200, 300 mM), glucose oxidase + glucose (GO+G), 3 µM DPI, 2 mM 3-AT, or mock/control infiltration. Raw data and sample sizes are provided in **Supplementary Table S4**. Statistical analysis was performed by one-way ANOVA followed by Tukey’s *post hoc* test (α = 0.05); significant differences are indicated by CLD.

Supplementary Figure 2 *B. cinerea* infection of Arabidopsis Col-0 and *rbohD* mutant. **(A)** Scheme of the three preincubation conditions used (8, 4 and 0 h; hi). **(B)** Relative lesion area in Col-0 and *rbohD* leaves 48 h after inoculation with conidia preincubated for 8, 4 or 0 h. Raw data and sample sizes are provided in **Supplementary Table S4**. Statistical significance was assessed using an unpaired t-test, *p<0.033, **p<0.002, ***p<0.001. **(C)** Representative images of infected leaves for each genotype and preincubation time, corresponding to the data in **(B)**.

Supplementary Figure 3 Effects of oxidative stress treatments on *B. cinerea* germ tube growth. **(A)** Germ tube length (% of control) of spores and pregerminated spores (“Germlings”) exposed to 10 mM H_2_O_2_ or 50 µM menadione. Raw data and sample sizes are provided in **Supplementary Table S4**. Statistical analysis was performed by one-way ANOVA followed by Tukey’s *post hoc* test (α = 0.05); significant differences are indicated by CLD. **(B)** Representative bright field micrographs of spores and germlings under different conditions.

Supplementary Figure 4 Cell death induced by different *B. cinerea* strains in Arabidopsis Col-0 leaf discs before and after H_2_O_2_ quantification. **(A)** Cell death, expressed as % chlorophyll autofluorescence lost, in leaf discs inoculated with *B. cinerea* WT, 18x or *bmp1* at 6 and 20 hpi. Raw data and sample sizes are provided in **Supplementary Table S4**. Statistical analysis was performed by one-way ANOVA followed by Tukey’s *post hoc* test (α = 0.05); significant differences are indicated by CLD. **(B)** Representative confocal images (chlorophyll autofluorescence) of mock- and strain-inoculated discs.

Supplementary Figure 5 PCR-based verification of *B. cinerea* ROS detoxification deletion mutants. Partially created with BioRender (https://www.biorender.com/).

Supplementary Figure 6 Growth phenotype of *srpk1* knockout mutants. Colonies of *B. cinerea* WT and three independent *srpk1* mutants (*srpk1-1*, *srpk1-2*, *srpk1-3*) grown on ME agar.

Supplementary Figure 7 Growth and developmental phenotypes of *B. cinerea* WT and oxidative stress-related mutants (*sod1, ccp1, cat2, ap1, ap1sod1, ap1sod1cat2*). **(A)** Relative growth rate (% of WT). **(B)** Relative sporulation (% of WT). **(C)** Relative sclerotia formation (% of WT). **(D)** Relative length of germination tube (% of WT). Raw data and sample sizes are provided in **Supplementary Table S4**. Statistical analysis was performed by one-way ANOVA followed by Tukey’s *post hoc* test (α = 0.05); significant differences are indicated by CLD.

Supplementary Figure 8 Gene expression profiles of Arabidopsis Col-0 and *rbohD* knockout mutant after *B. cinerea* infection. RT-qPCR analysis of *PR1*, *PDF1.2* and *WRKY33* expression at 18, 24, 48 and 72 hpi. Gene expression values are shown as log_2_ fold changes relative to mock-treated plants, with bars representing the mean ± SD. Raw data and sample sizes are provided in **Supplementary Table S4**. Statistical analysis was performed by two-way ANOVA followed by Šidák’s multiple comparisons test (α = 0.05); significant differences are indicated by CLD.

Supplementary Figure 9 Quantitative comparison of the transcriptomes of Col-0 and *RBOHD^MUT^*. **(A)** Principal component analysis of the 12 RNA-seq samples (variance-stabilized counts). Green, Col-0; black, *RBOHD^MUT^*; open symbols, mock; filled symbols, *B. cinerea* at 48 hpi; symbol shape, replicate set. **(B)** Correlation of the infection-induced changes in gene expression (log_2_FC, *B. cinerea* vs mock) in Col-0 and *RBOHD^MUT^* (22,020 genes; dashed line, identity). **(C)** Overlap of the genes differentially expressed after infection in Col-0 and *RBOHD^MUT^* (FDR ≤ 0.05, |FC| ≥ 1.5). **(D)** Volcano plot of the comparison *RBOHD^MUT^* vs Col-0 at 48 hpi; the 15 DEGs (**Supplementary Table S2**) are shown in red; dashed lines indicate FDR = 0.05 and |FC| = 1.5. **(E)** GSEA (GO biological process) of *RBOHD^MUT^* vs Col-0 at 48 hpi. Each dot is a gene set; positive normalized enrichment scores indicate higher expression in *RBOHD^MUT^*. Red, FDR < 0.05; blue, selected defense-, hormone- and ROS-related terms; orange, photosynthesis; dashed line, FDR = 0.05.

Supplementary Table 1 Primers used in this work. sgRNA sequences for CRISPR/Cas9-mediated gene deletion and primers for PCR verification of the B. cinerea mutants (**Figure S5**), and RT-qPCR primers for Arabidopsis genes (**Figure 7C**, **Figure S8**). HKG, housekeeping (reference) gene; GOI, gene of interest.

Supplementary Table 2 Differentially expressed genes (DEGs) between RBOHDMUT and Col-0 plants 48 hours after B. cinerea infection. Positive log2 fold-change values indicate higher expression in RBOHDMUT relative to Col-0, negative values lower expression in RBOHDMUT. DEGs were defined by an FDR-adjusted p-value ≤ 0.05 and a fold change |FC| ≥ 1.5 (|log2FC| ≥ 0.585). ID, row number in the heat map of **Figure 7A** (right); see also **Figure S9D**.

Supplementary Table 3 Differentially expressed redox-related genes in Arabidopsis Col-0 and RBOHDMUT leaves 48 hours after B. cinerea infection. Positive log2 fold-change values indicate higher transcript abundance in infected plants relative to mock-treated controls, negative values lower abundance. Genes are listed if they met the DEG criteria (FDR ≤ 0.05 and |FC| ≥ 1.5, i.e. |log2FC| ≥ 0.585) in both genotypes. AsA, ascorbate-dependent redox network; Dis, ROS dismutation; GSH, glutathione-dependent redox network; Trx, thioredoxin-dependent redox network. Compartment(s), predicted subcellular localization.

Supplementary Table 4 Raw data and sample sizes of **Figures 1**–**7**, **S1–S4**, **S7–S9** (one sheet per figure; sheets marked “(raw)” contain non-normalized lesion areas). The **Figure 7A** sheet contains the complete differential expression results (CLC Genomics Workbench) for all 27,655 annotated genes, and the **Figure 7C** sheet includes the RT-qPCR primer efficiencies.
