# Supplementary material for: Botrytis cinerea uses effective detoxification mechanisms to overcome the plant RBOHD-dependent extracellular oxidative burst
Source: Front Plant Sci. 2026 Oct 16;17:1969467. doi: 10.3389/fpls.2026.1969467 (PMC13719456; doi:10.3389/fpls.2026.1969467)
Supplement: Supplementary file 1 [file Table1.docx]

**Table S1. Primers used in this work.** sgRNA sequences for CRISPR/Cas9-mediated gene deletion and primers for PCR verification of the *B. cinerea* mutants (Figure S5), and RT-qPCR primers for Arabidopsis genes (Figure 7C, Figure S8). HKG, housekeeping (reference) gene; GOI, gene of interest.

| ***Botrytis cinerea*** | | | | |
| --- | --- | --- | --- | --- |
| **Bcin code** | **Gene name** | **Primer name** | **Primer sequence (5’ → 3’)** | **Application** |
| Bcin08g03050 | *BcSrpk1* | Srpk1-L1 | GCCAACTCGCTACACGCTCG | sgRNA for CRISPR-Cas |
|  |  | Srpk1-R1 | AGAACGAATGGATTACTAGG |  |
|  |  | BcSrpk1-Outer_Fw | TTGCCCATCTCGTCCAATCC | Genetic confirmation of transformant |
|  |  | BcSrpk1-Outer_Rv | CCAGCACAGGAGTCAAGGAG |  |
|  |  | BcSrpk1-Inner_Fw | ATCGGAAATCCCTGGCAGTG |  |
|  |  | BcSrpk1-Inner_Rv | CTGGTAAGGCCCAATGTCGT |  |
| Bcin01g09360 | *BcCcp1* | Ccp1-L1 | AGAAAATGGAAAGTACAGAG | sgRNA for CRISPR-Cas |
|  |  | Ccp1-R1 | GGTGAATTAAGGTATCTAGA |  |
|  |  | BcCcp1-Outer_Fw | TGCTTGCTTGAACCCTTCGT | Genetic confirmation of transformant |
|  |  | BcCcp1-Outer_Rv | CAATCTCCCTCCTACCCCTCA |  |
|  |  | BcCcp1-Inner_Fw | CACCTTTCAGATCCGCCACT |  |
|  |  | BcCcp1-Inner_Rv | CCATGGGTGCTTAGCTGTGA |  |
| Bcin03g03390 | *BcSod1* | Sod1-L1 | GGTTGTAAGGAACTACGTAG | sgRNA for CRISPR-Cas |
|  |  | Sod1-R1 | AATGAAAGTGGGCTTGCTCG |  |
|  |  | BcSod1-Outer_Fw | CTGGGTGCCGTACGAAATGT | Genetic confirmation of transformant |
|  |  | BcSod1-Outer_Rv | GCAAGCACAACTCCCTCCAT |  |
|  |  | BcSod1-Inner_Fw | GCGTTACCGGTCTTCTTGGA |  |
|  |  | BcSod1-Inner_Rv | GACTCCAAGATCTCCGGCAC |  |
| Bcin11g06450 | *BcCat2* | Cat2-L1 | GATGCAATCATCCTACCTTG | sgRNA for CRISPR-Cas |
|  |  | Cat2-R1 | TTAAGTGACGATCGAATTCA |  |
|  |  | BcCat2-Outer_Fw | AGCCTTTTATCTGCCGGGTC | Genetic confirmation of transformant |
|  |  | BcCat2-Outer_Rv | CCGAAGTGTTTTCCCGCAAG |  |
|  |  | BcCat2-Inner_Fw | ACCCCACCAAGATCCTTCCT |  |
|  |  | BcCat2-Inner_Rv | TTCAAAACTGAAGCGACGCG |  |
| Bcin13g03000 | *BcAp1* | Ap1-L1 | AGTGGGATTTGGTTTCGAGG | sgRNA for CRISPR-Cas |
|  |  | Ap1-R1 | TAGGTAGTTAAGTGAATAGG |  |
|  |  | BcAp1-Outer_Fw | CCCCTTTCGAACAAGTTGCG | Genetic confirmation of transformant |
|  |  | BcAp1-Outer_Rv | AACCTGCAATGACCCTACCG |  |
|  |  | BcAp1-Inner_Fw | AAGAAGCCCGGTAGAAAGCC |  |
|  |  | BcAp1-Inner_Rv | GCCGATCTGAGCTGGAGTTT |  |
| ***Arabidopsis thaliana*** | | | | |
| **AGI code** | **Gene name** | **Primer name** | **Primer sequence (5’ → 3’)** | **Application** |
| AT3G18780 | *AtActin2* | ACT2_Fw | TCTCAGGTATCGCTGACCGT | HKG qPCR |
|  |  | ACT2_Rv | CCTGCCTCATCATACTCGGC |  |
| AT2G14610 | *AtPR1* | PR1_Fw | GCAGAACAACTAAGAGGCAAC | GOI1 qPCR |
|  |  | PR1_Rv | ACCACAAACTCCATTGCAC |  |
| AT5G44420 | *AtPDF1.2* | PDF1.2_Fw | ACGCACCGGCAATGGTGGAA | GOI2 qPCR |
|  |  | PDF1.2_Rv | TGCATGATCCATGTTTGGCTC |  |
| AT2G38470 | *AtWRKY33* | wrky33_Fw | CTCGTGGTAGCGGTTACGCC | GOI3 qPCR |
|  |  | wrky33_Rv | CCTTTGCTCTAGAGAATCCACC |  |
| AT3G26830 | *AtPAD3* | PAD3_Fw | GTGGCGCTTCTCCAATACGG | GOI4 qPCR |
|  |  | PAD3_Rv | ACTCCACTCGTCACCGAAGG |  |
| AT3G23240 | *AtERF1* | ERF1_Fw | TCCCCATTCTCCGGCTTCTC | GOI5 qPCR |
|  |  | ERF1_Rv | TTTGTTGCGTGGACTGCTCG |  |
| AT2G39660 | *AtBIK1* | BIK1_Fw | GCTCAGAAGACGGAAGGGGA | GOI6 qPCR |
|  |  | BIK1_Rv | AGCCACCTTCTCCGATCACA |  |
| AT3G52430 | *AtPAD4* | PAD4_Fw | CGAGACGAGTGAGTTGCAAG | GOI7 qPCR |
|  |  | PAD4_Rv | ACGTATGTAATCCCGGCGAT |  |
