# Supplementary material for: Botrytis cinerea uses effective detoxification mechanisms to overcome the plant RBOHD-dependent extracellular oxidative burst
Source: Front Plant Sci. 2026 Oct 16;17:1969467. doi: 10.3389/fpls.2026.1969467 (PMC13719456; doi:10.3389/fpls.2026.1969467)
Supplement: Supplementary file 2 [file Table2.docx]

**Table S2. Differentially expressed genes (DEGs) between *RBOHD^MUT^* and Col-0 plants 48 hours after *B. cinerea* infection**. Positive log₂ fold-change values indicate higher expression in RBOHD^MUT^ relative to Col-0, negative values lower expression in RBOHD^MUT^. DEGs were defined by an FDR-adjusted p-value ≤ 0.05 and a fold change |FC| ≥ 1.5 (|log₂FC| ≥ 0.585). ID, row number in the heat map of Figure 7A (right); see also Figure S9D.

| **ID** | **AGI code** | **Name** | **log₂ Fold change** | **FDR** | **Functional annotation** | **Description** |
| --- | --- | --- | --- | --- | --- | --- |
| **9** | **AT5G24240** | PI4Kγ3 (PI4Kc3) | 4.295 | 7.59E-11 | Phosphatidylinositol 4-kinase γ-like protein | Nuclear PI4Kγ-like protein; overexpression delays flowering. |
| **10** | **AT5G65080** | MAF5 / AGL68 | 3.188 | 1.10E-11 | MADS-box transcription factor | Represses flowering; induced by vernalization. |
| **14** | **AT5G65070** | MAF4 / AGL69 | 1.508 | 0.049 | MADS-box transcription factor | *FLC* paralog delaying flowering; down-regulated after vernalization. |
| **13** | **AT2G38823** | — | 1.206 | 0.001 | Unknown protein | Predicted protein of unknown function. |
| **12** | **AT3G56210** | — | 0.952 | 3.36E-07 | ARM-repeat superfamily protein | Predicted ARM-repeat protein; uncharacterized. |
| **11** | **AT4G32551** | LUG (LEUNIG) | 0.888 | 6.38E-06 | Transcriptional corepressor | Regulates floral organ identity; represses AGAMOUS. |
| **15** | **AT1G78210** | — | 0.735 | 0.025 | α/β-hydrolase superfamily protein | Predicted hydrolase; unknown role. |
| **5** | **AT4G08870** | ARGAH2 | -0.841 | 0.001 | Arginase (arginine amidohydrolase) | *B. cinerea*-responsive arginase; JA-induced. |
| **2** | **AT1G15520** | ABCG40 / PDR12 | -0.974 | 0.001 | ATP-binding cassette transporter G40 | ABA exporter involved in stress responses. |
| **4** | **AT4G13540** | ADR | -1.049 | 0.007 | Myristoylated protein | Expressed in flowers; affects ROS during anther opening. |
| **8** | **AT1G09240** | NAS3 | -1.111 | 0.011 | Nicotianamine synthase | Synthesizes nicotianamine for metal homeostasis. |
| **6** | **AT3G21670** | NPF6.4 | -1.33 | 0.047 | Nitrate/peptide transporter (NPF) | Nitrate/peptide transporter (NPF family). |
| **1** | **AT5G41830** | — | -1.569 | 0.028 | RNI-like superfamily protein | Reticulon-related membrane protein; uncharacterized. |
| **3** | **AT5G59940** | — | -1.856 | 6.31E-08 | C1-domain family protein | Cys/His-rich C1-domain signaling protein. |
| **7** | **AT4G36430** | — | -2.952 | 0.049 | Peroxidase superfamily protein | Class III peroxidase; likely in ROS detoxification. |
