# Supplementary material for: Botrytis cinerea uses effective detoxification mechanisms to overcome the plant RBOHD-dependent extracellular oxidative burst
Source: Front Plant Sci. 2026 Oct 16;17:1969467. doi: 10.3389/fpls.2026.1969467 (PMC13719456; doi:10.3389/fpls.2026.1969467)
Supplement: Supplementary file 3 [file Table3.docx]

**Table S3. Differentially expressed redox-related genes in Arabidopsis Col-0 and *RBOHD^MUT^* leaves 48 hours after *B. cinerea* infection**. Positive log₂ fold-change values indicate higher transcript abundance in infected plants relative to mock-treated controls, negative values lower abundance. Genes are listed if they met the DEG criteria (FDR ≤ 0.05 and |FC| ≥ 1.5, i.e. |log₂FC| ≥ 0.585) in both genotypes. AsA, ascorbate-dependent redox network; Dis, ROS dismutation; GSH, glutathione-dependent redox network; Trx, thioredoxin-dependent redox network. Compartment(s), predicted subcellular localization.

| **AGI code** | **Name** | **Col-0**  **(*Botrytis* VS Mock)** | | ***RBOHD^MUT^***  **(*Botrytis* VS mock)** | | **Functional annotation** | **Compartment(s)** | **Redox network** |
| --- | --- | --- | --- | --- | --- | --- | --- | --- |
|  |  | **log₂ Fold change** | **FDR** | **log₂ Fold change** | **FDR** |  |  |  |
| **AT1G69880** | TH8 | 10.814 | 1.59E-40 | 11.535 | 2.58E-22 | Thioredoxin h8 | Cytosol | Trx |
| **AT1G60740** | - | 8.082 | 1.06E-22 | 7.703 | 1.76E-33 | PRXIID | Cytosol | GSH; Trx |
| **AT2G31570** | GPXL2 | 4.015 | 3.93E-71 | 4.047 | 7.85E-64 | Glutathione peroxidase-like | Cytosol | Trx |
| **AT1G75270** | DHAR2 | 3.986 | 1.82E-106 | 4.231 | 6.52E-180 | Dehydroascorbate Reductase 2 | Cytosol | GSH; AsA |
| **AT1G45145** | TRX5 | 3.093 | 3.39E-25 | 2.935 | 1.05E-31 | Thioredoxin h5 | Cytosol | Trx |
| **AT1G08830** | CSD1 | 2.923 | 1.24E-22 | 3.481 | 6.95E-33 | Copper/zinc superoxide dismutase 1 | Cytosol | Dis |
| **AT5G03630** | MDAR2 | 2.739 | 4.19E-22 | 2.676 | 4.72E-29 | Monodehydroascorbate Reductase 2 | Cytosol | AsA |
| **AT1G07890** | APX1 | 2.694 | 1.95E-20 | 2.429 | 2.79E-23 | Ascorbate Peroxidase 1 | Cytosol | AsA |
| **AT5G40370** | GRXC2 | 2.473 | 4.50E-29 | 2.528 | 9.81E-32 | Glutaredoxin C2 | Cytosol | GSH |
| **AT1G63460** | GPX8 | 1.228 | 5.28E-13 | 1.006 | 4.71E-10 | Glutathione peroxidase-like 8 | Cytosol | Trx |
| **AT5G39950** | TRX2 | 1.175 | 3.30E-13 | 0.914 | 6.55E-10 | Thioredoxin h2 | Cytosol | Trx |
| **AT1G65980** | TPX1 | 0.968 | 3.17E-05 | 0.922 | 5.25E-05 | PRXIIB | Cytosol | GSH; Trx |
| **AT2G17420** | NTRA | 2.695 | 3.04E-35 | 2.784 | 1.22E-38 | NADPH-dependent thioredoxin reductase A | Cytosol, mitochondrion | Trx |
| **AT4G35460** | NTRB | 2.166 | 3.52E-36 | 2.253 | 2.32E-45 | NADPH-dependent thioredoxin reductase B | Cytosol, mitochondrion | Trx |
| **AT3G24170** | GR1 | 3.609 | 5.45E-58 | 3.744 | 1.54E-73 | Glutathione reductase 1 | Cytosol, peroxisome | GSH; AsA |
| **AT2G48150** | GPXL4 | 3.100 | 1.59E-08 | 1.491 | 8.12E-04 | Glutathione peroxidase-like 4 | Cytosol, plasma membrane | Trx |
| **AT3G08710** | TRHX9 | 2.534 | 3.04E-63 | 2.657 | 2.36E-46 | Thioredoxin h9 | Cytosol, plasma membrane | Trx |
| **AT5G27380** | GSH2 | 3.566 | 3.28E-46 | 3.544 | 9.66E-44 | Glutathione synthetase 2 | Cytosol, plastid | GSH |
| **AT3G10920** | MSD1 | 2.062 | 4.67E-28 | 2.042 | 6.25E-30 | MnSOD 1 | Mitochondrion | Dis |
| **AT4G11600** | GPXL6 | 1.873 | 3.81E-19 | 1.919 | 7.76E-23 | Glutathione peroxidase-like 6 | Mitochondrion | Trx |
| **AT3G06050** | PRXIIF | 1.454 | 3.34E-20 | 1.586 | 1.02E-26 | Peroxiredoxin II F | Mitochondrion | Trx |
| **AT2G35010** | TRXO1 | 1.113 | 7.03E-10 | 0.908 | 6.87E-07 | Thioredoxin o1 | Mitochondrion | Trx |
| **AT1G20630** | CAT1 | 3.691 | 3.06E-65 | 3.554 | 4.33E-57 | Catalase 1 | Peroxisome | Dis |
| **AT3G52880** | MDAR1 | 1.939 | 8.63E-17 | 2.022 | 9.44E-19 | Monodehydroascorbate Reductase 1 | Peroxisome | AsA |
| **AT3G27820** | MDAR4 | 1.056 | 1.30E-10 | 0.938 | 1.96E-08 | Monodehydroascorbate Reductase 4 | Peroxisome | AsA |
| **AT4G35090** | CAT2 | -3.386 | 6.40E-45 | -3.581 | 6.27E-67 | Catalase 2 | Peroxisome | Dis |
| **AT1G20620** | CAT3 | -1.902 | 1.20E-28 | -1.860 | 3.01E-30 | Catalase 3 | Peroxisome | Dis |
| **AT4G23100** | GSH1 | 4.392 | 5.35E-63 | 4.347 | 1.03E-63 | Glutamate-cysteine ligase | Plastid | GSH |
| **AT4G28730** | GrxC5 | 1.681 | 5.03E-11 | 1.747 | 1.03E-12 | Glutaredoxin C5 | Plastid | GSH |
| **AT1G76760** | TY1 | 1.601 | 1.41E-07 | 1.776 | 2.21E-09 | Thioredoxin y1 | Plastid | Trx |
| **AT4G25130** | MSRA4 | 1.396 | 2.15E-20 | 1.433 | 1.44E-20 | Methionine sulfoxide reductase A4 | Plastid | Trx |
| **AT2G28190** | CSD2 | 1.254 | 7.13E-08 | 2.298 | 1.27E-24 | Copper/zinc superoxide dismutase 2 | Plastid | Dis |
| **AT2G41680** | NTRC | 0.808 | 4.34E-08 | 0.904 | 2.85E-10 | NADPH-dependent thioredoxin reductase C | Plastid | Trx |
| **AT2G38270** | GRXS16 | 0.751 | 2.29E-06 | 0.721 | 1.44E-06 | Glutaredoxin S16 | Plastid | GSH |
| **AT4G21860** | MSRB2 | -0.782 | 1.25E-06 | -0.959 | 2.86E-11 | Methionine sulfoxide reductase B2 | Plastid | Trx |
| **AT3G54900** | GRXS14 | -0.860 | 1.05E-07 | -0.839 | 1.20E-07 | Glutaredoxin S14 | Plastid | GSH |
| **AT2G04700** | - | -0.967 | 1.05E-10 | -1.116 | 4.92E-12 | Ferredoxin-dependent thioredoxin reductase, FTRB, catalytic subunit | Plastid | Trx |
| **AT3G06730** | TRX z | -1.145 | 2.51E-08 | -0.915 | 4.54E-05 | Thioredoxin z | Plastid | Trx |
| **AT5G23440** | FTRA1 | -1.213 | 4.53E-15 | -1.107 | 2.08E-10 | Ferredoxin-dependent thioredoxin reductase, FTRA1, variable subunit | Plastid | Trx |
| **AT1G03680** | THM1 | -1.531 | 6.74E-20 | -1.779 | 3.86E-25 | Thioredoxin m1 | Plastid | Trx |
| **AT5G06290** | 2CPrxB | -1.603 | 8.24E-23 | -1.808 | 9.93E-24 | 2-Cys-Peroxiredoxin B | Plastid | Trx |
| **AT3G55040** | GSTL2 | -1.711 | 2.42E-21 | -1.594 | 4.78E-12 | Glutathione S-Transferase Lambda 2 | Plastid | GSH |
| **AT3G11630** | 2CPrxA | -1.769 | 2.22E-20 | -1.724 | 8.00E-20 | 2-Cys-Peroxiredoxin A | Plastid | Trx |
| **AT2G25080** | GPX1 | -2.050 | 3.15E-19 | -2.066 | 1.89E-20 | Glutathione peroxidase-like 1 | Plastid | Trx |
| **AT5G16710** | DHAR3 | -2.083 | 5.43E-28 | -2.178 | 3.11E-31 | Dehydroascorbate Reductase 3 | Plastid | GSH; AsA |
| **AT5G06690** | WCRKC1 | -2.516 | 2.35E-30 | -2.688 | 3.77E-40 | TRX-LIKE 2.1 | Plastid | Trx |
| **AT5G16400** | TRXF2 | -2.529 | 4.18E-34 | -2.644 | 1.25E-30 | Thioredoxin f2 (Trxf2) | Plastid | Trx |
| **AT1G77490** | TAPX | -3.059 | 2.59E-61 | -3.166 | 4.18E-78 | thylakoidal Ascorbate peroxidase | Plastid | AsA |
| **AT3G02730** | TRXF1 | -3.317 | 3.21E-70 | -3.159 | 8.66E-55 | Thioredoxin f1 | Plastid | Trx |
| **AT3G26060** | PRXQ | -4.300 | 1.10E-53 | -4.339 | 6.85E-42 | Peroxiredoxin Q | Plastid | Trx |
| **AT4G08390** | SAPX | 2.731 | 9.52E-40 | 2.792 | 5.45E-66 | Stromal Ascorbate Peroxidase | Plastid, mitochondrion | AsA |
